# Antiparasitic efficacy of geraniol from Apiaceae family in scabies treatment

**DOI:** 10.1038/s41598-025-97702-z

**Published:** 2025-05-15

**Authors:** Iman S. A. Khallaf, Lourin G. Malak, Soad A. L. Bayoumi, Salwa F. Farag, Ahmed M. Sayed, Sara A. Mohamed, Asmaa A. E. Nasr, Radwa Y. Ibrahim, Eman Maher Zahran, Gerhard Bringmann, Usama Ramadan Abdelmohsen

**Affiliations:** 1 Pharmacognosy and Natural Products Department, Faculty of Pharmacy, Menoufia University, Shibin Elkom, 32511 Egypt; 2 Pharmacognosy Department, Faculty of Pharmacy, Assiut University, Assiut, 71526 Egypt; 3 Pharmacognosy Department, College of Pharmacy, Taif University, P.O. Box 11099, 21944 Taif, Saudi Arabia; 4 Department of Pharmacognosy, College of Pharmacy, Almaaqal University, Basrah, 61014 Iraq; 5 Department of Pharmacognosy, Faculty of Pharmacy, Nahda University, Beni-Suef, 62513 Egypt; 6 Department of Parasitology, Faculty of Veterinary Medicine, Assiut University, Assiut, 71515 Egypt; 7 Department of Poultry Diseases, Animal Health Research Institute, Assiut Regional Laboratory, Agricultural Research Center (ARC), Assiut, Egypt; 8 Department of Medical Parasitology, Faculty of Medicine, Assiut University, Assiut, Egypt; 9 Department of Pharmacognosy, Faculty of Pharmacy, Deraya University, 7 Universities Zone, New Minia City, 61111 Egypt; 10 Institute of Organic Chemistry, University of Würzburg, Am Hubland, 97074 Würzburg, Germany; 11 Deraya Center for Scientific Research, Deraya University, 7 Universities Zone, New Minia City, 61111 Egypt

**Keywords:** Acaricidal activity, Acetylcholinesterase, Apiaceae, Geraniol, In silico analysis

## Abstract

*Sarcoptic mange*, commonly known as scabies, is a highly contagious skin condition caused by the burrowing mite Sarcoptes scabiei (Astigmata: Sarcoptinae). This parasitic disease significantly impacts livestock and human health, particularly in underserved regions. Current treatments rely on synthetic acaricides like permethrin and ivermectin, which suffer from limitations such as toxicity, resistance development, and environmental contamination. Essential oils from Apiaceae plants represent a promising natural alternative. This study reviewed 122 volatile constituents from Apiaceae plants and conducted comprehensive in silico analyses to identify potential antiparasitic agents. Geraniol emerged as a potent acaricidal candidate due to its strong binding affinity to acetylcholinesterase (AChE) (docking score: − 7.85 kcal/mol). In vitro testing revealed geraniol achieved a 100% mite mortality rate at concentrations as low as 6.25% within 15 min (LT_50_ = 9.5 min). In vivo studies using scabies-infected rabbits demonstrated that geraniol-treated animals exhibited complete clinical recovery by two weeks post-treatment, with disappearance of crusts, itching, and skin thickening. Histopathological examination showed near-complete skin regeneration with minimal inflammatory infiltrates, in contrast to control groups which exhibited severe lesions and active mite presence. Furthermore, geraniol-treated rabbits displayed new hair growth and improved general condition, with no observed adverse effects. These findings highlight geraniol’s potential as a safe, effective, and eco-friendly treatment for scabies, offering a 100% improvement in clinical and histological recovery within two weeks. Further research should focus on optimizing delivery systems and evaluating its efficacy in human clinical trials.

**Supplementary Information:**

The online version contains supplementary material available at 10.1038/s41598-025-97702-z.

## Introduction

Parasitic diseases are considered as a widespread health problem in rural tropical areas where humidity and high temperatures promote the growth of parasites. Many parasites affect populations with low standards of living and poor hygiene and sanitation^1–3^. Human parasitic diseases can be caused by endoparasites (protozoa and helminth worms) and ectoparasites (fleas, lice, ticks, and mites)^1^. The *Sarcoptes scabiei* var. *hominis* itch mite is an ectoparasite causing a contagious parasitic skin infestation in humans called scabies^4^. It develops intense pruritus and skin rash as a result of the immune reaction caused by burrowing the mites into the skin epidermis and laying eggs^5^. Scabies causes impetigo (secondary bacterial skin infection), which leads to many complications such as streptococcal septicemia, rheumatic fever, and chronic heart and renal diseases^6,7^. It is estimated by the World Health Organization (WHO) that 200 million people worldwide are currently infested^8^ with high prevalence in overcrowded, poor regions and during wars^9^. WHO has recognized scabies as a neglected tropical disease in 2017^8,10^.

Ivermectin and permethrin are commonly used for the treatment of scabies in addition to sulfur compounds, γ-benzene hexachloride (lindane), and benzyl benzoate. However, these drugs show adverse effects, and many studies have reported resistance to them^11,12^. This prompted the development of effective and safe alternative treatments to appropriately manage scabies. Nature provides humanity with several metabolites for treatment of parasitic diseases either for human or animals^13–18^. Several studies have shown that essential oils (EOs) derived from plants in addition to some plant extracts are being used in the treatment of scabies, such as tea tree oil, *Lippia multiflora* oil, *Azadirachta indica* oil, *Curcuma long* oil and coconut seed extract^11,12,19^.

EOs are natural complex mixtures of volatile organic substances; they are extracted by hydro- or steam-distillation from different parts of the plants^20^. EOs of many plants contain small lipophilic compounds which show antiparasitic properties by disturbing their biomembranes^21^. In addition, the hydrophobicity of the EO components and their low density promote their intracellular targeting into parasites. The antiparasitic activity of plant EOs may be due to their immunomodulatory effects through enhancing nitric oxide (NO) production in macrophages. NO protects macrophages against parasitic infections. Moreover, EOs have anti-inflammatory activity, which protects the host from NO damaging effects^22^. Another mode of action of antiparasitic activity of EOs has been suggested. The hydrophobic EOs and their components bind to the hydrophobic site of cruzain, a cysteine protease enzyme, inhibiting it and blocking the life cycle of the parasite^19,23,24^.

The plant family Apiaceae, formerly known as Umbelliferae, comprises approximately 434 genera and 3780 species, distributed in the tropical areas. Many studies have reported biological activities of Apiaceae plants such as antimicrobial, antitumor, analgesic, radical scavenging, diuretic, anti-inflammatory, anti-obesity, and insecticidal properties. Besides, most of these plants are used traditionally to treat respiratory, reproductive, and gastrointestinal tract disorders^25,26^. Plants belonging to this family are rich in different bioactive compounds such as coumarins, polyacetylenes, terpenoids and triterpenoid saponins, flavonoids, and steroids. In addition, Apiaceae plants are characterized by the presence of EOs secreted by schizogenous oil ducts located in their different organs and responsible for their distinctive flavors. The volatile oil components isolated from different Apiaceae plants are listed in Table S1. These EOs exhibit insecticidal, insect repellents, antifeedant and growth regulators activities^25–27^. In addition, EOs found in members of family Apiaceae display interesting antiparasitic activity. As an example, the EO of *Anethum graveolens* showed anthelmintic activity against *Haemonchus contortus*, when compared with thybendazole and levamisole as positive controls^28^. The EO of *Pimpinella anisum* was found to possess acaricidal activity on adult *Dermanyssus gallinae*, the poultry red mite, with an LC_50_ value of 47.5 μg/mL when compared with permethrin as the positive control and against *Tetranychus urticae*^29,30^. *Apium graveolens* EO showed antiparasitic activity against the larvae of *Aedes aegypti* in addition to repelling mosquitoes when mixed with 5% vanillin^31^. The EO of *Ferula macrecolea* had antiparasitic activity against protoscoleces of *Echinococcus granulosus*^32^. The EO of *Foeniculum vulgare* exerted moderate anthelmintic activity against adult worms of *Schistosoma mansoni*^33^. In addition, it showed acaricidal activity against *Rhipeciphalus annulatus*, *Dermatophagoides pteronyssinus*, and *Dermatophagoides farina* and it exerted larvicidal activity against the Asian vector of malaria*, Anopheles stephensi*^34–36^. The EO of *Petroselinum crispum* exhibitted insecticidal activity against *Trialeurodes vaporariorum* adult pest^37^. The EO of *Carum carvi* showed antiprotozoal activity against *Plasmodium falciparum*^38^, while the EO of *Cuminum cyminum* possessed antiparasitic activity against the insects *Anopheles gambiae*, *Tribolium confusum*, and *Ephestia kuehniella*^38,39^. *Coriandrum sativum* EO exerted antiparasitic activity against the insects *Tribolium confusum*, *Callosobruchus maculatus*, *Aedes albopictus*, and *Aedes aegypti* and against the nematodes *Bursaphelenchus xylophilus, Haemonchus contortus, Trichostrongylus colubriformis*, *Trichostrongylus axei*, *Trichostrongylus vitrines*, and* Teladorsagia circumcincta*^38,40^. Different antiparasitic activities of screened compounds are listed in Table S2.

Linalool, the major constituent of *Coriandrum sativum* oil, showed strong anthelmintic activity against *Trichostrongylus colubriformis* nematodes, *Trichostrongylus axei*, *Teladorsagia circumcincta*, *Trichostrongylus vitrines*, and *Haemonchus contortus* and exhibited a synergistic anthelmintic effect when combined with coriander EO^40^. (*R*)-Carvone and D-limonene, the components of EO of *Carum carvy* showed strong fumigant toxicity against *Sitophilus zeamais* and *Tribolium castaneum* adults, with LC_50_ values of 2.76 and 48.18 mg/L for *S*. *zeamais* and LC_50_ = 1.96 and 19.10 mg/L for *T. castaneum*, respectively. This makes the EO of *Carum carvi* to have antiparasitic activity against both *S. zeamais* and *T. castaneum* adults (LD_50_ values of 3.07 and 3.29 μg/adult respectively)^38,41^. (7,10) ( +)-Limonene epoxide exerted antischistosomal activity against *Schistosoma mansoni* adult worms^42^. The main component of *Foeniculum vulgare*, *trans*-anethole, exhibited acaricidal activity against *Rhipeciphalus annulatus*^34^.

Several studies reported the activity of EOs and their components against scabies (Table S3), but only few are found dealing with members of the Apiaceae family, which initiated us to carry out this work.

**Fig. 1:**
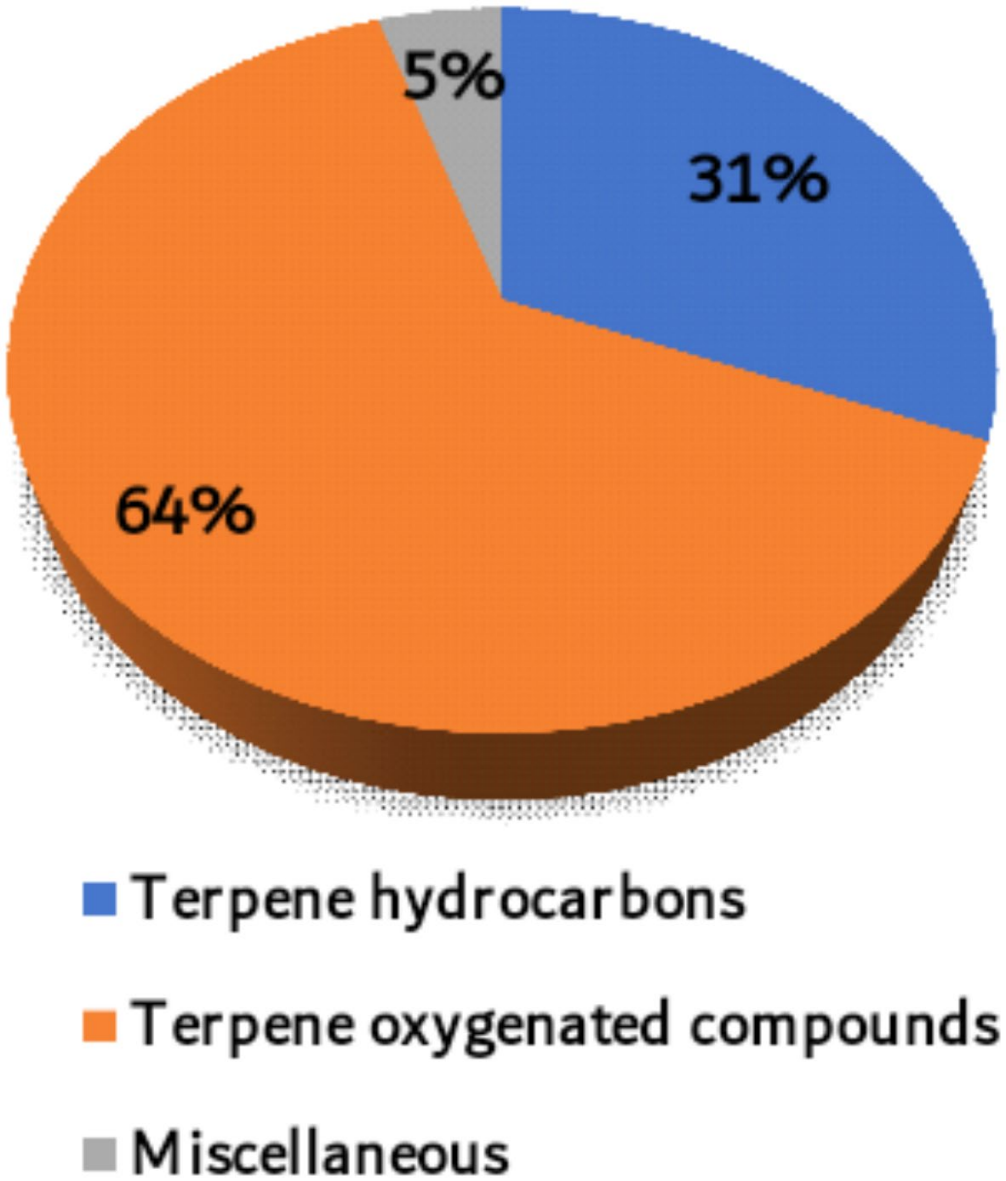
Chemistry of the investigated 122 volatile components identified from members of the Apiaceae family.

**Fig. 2:**
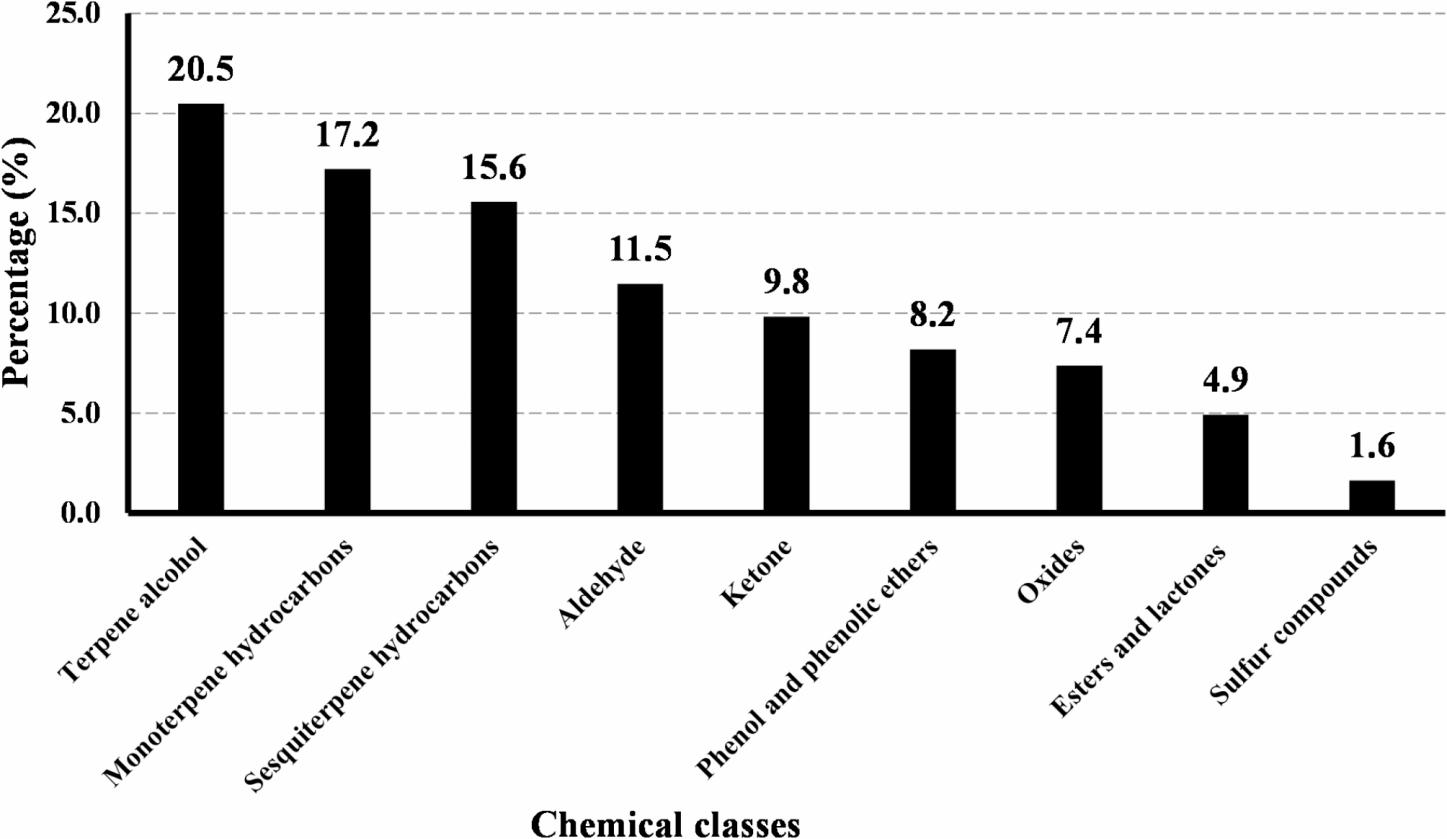
Percentage of the different chemical classes of the selected terpene compounds.

We examined the main volatile oil components identified in Apiaceae plants over the past years (until June 2024). From this research, we compiled a list of 122 key components, which can be found in Table S1. Chemically, these components are classified into terpene hydrocarbons (38 compounds), terpene oxygenated compounds (78 compounds) and miscellaneous compounds (six compounds), hence representing 31, 64, and 4.9% respectively, of the total number of compounds (Fig. 1). The terpene hydrocarbon compounds are classified into monoterpenes (19 compounds, 15.6%) and sesquiterpene hydrocarbons (19 compounds, 15.6%). On the other hand, oxygenated terpene compounds are classified into terpene alcohols (25 compounds, 20.5%), aldehydes (14 compounds, 11.5%), ketones (12 compounds, 9.8%), phenol and phenolic ether components (ten compounds, 8.2%), oxides (nine compounds, 7.4%), esters and lactones (six compounds, 4.9%) and sulfur-containing compounds (two compounds, 1.6%). Miscellaneous compounds are (six compounds, 4.9%) (Figs. 1 and 2).

**Fig. 3:**
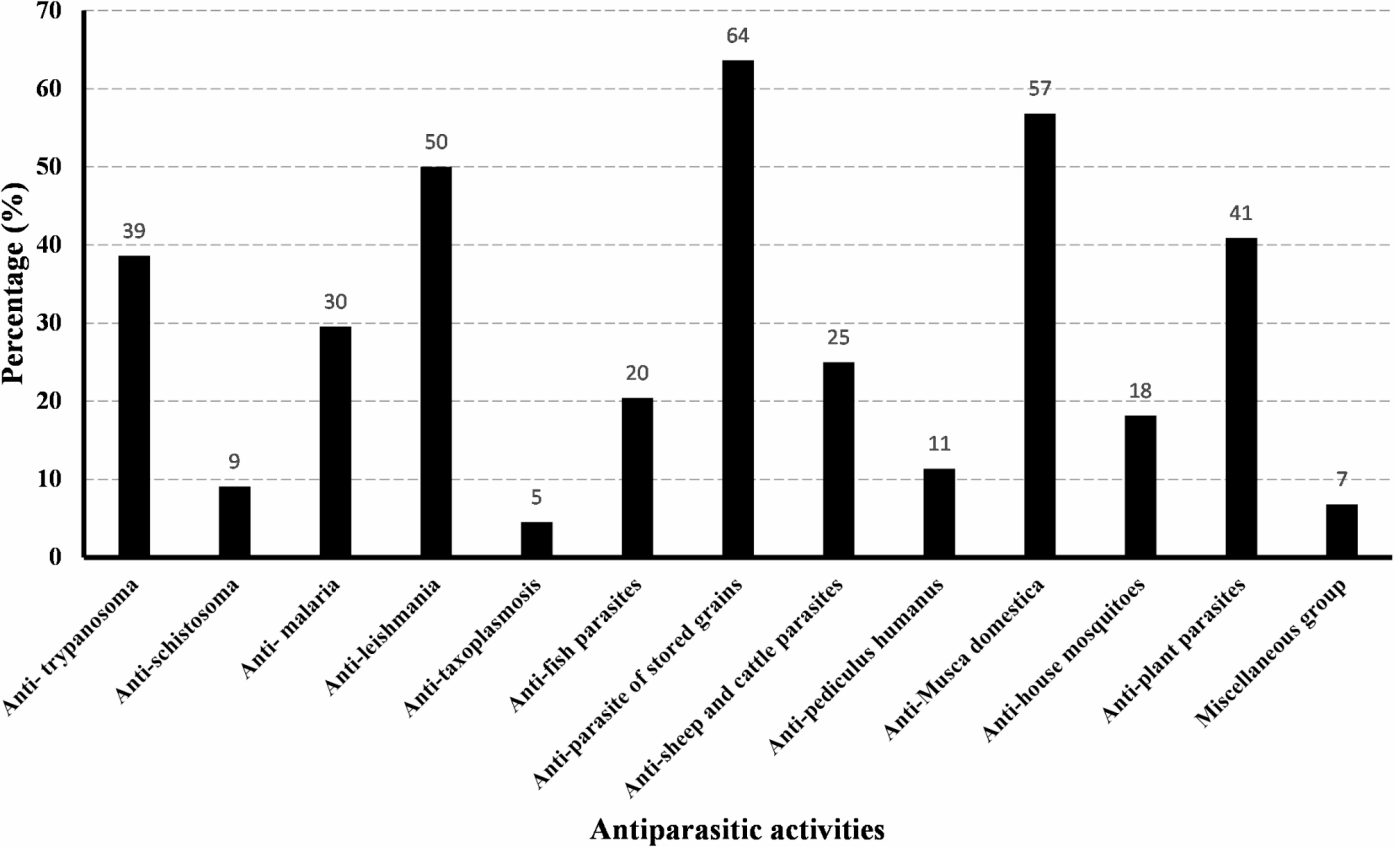
Different antiparasitic activities of the investigated compounds.

Among all reported compounds, only 44 compounds were reported to exhibit antiparasitic activity (Table S2). These compounds are: β-myrcene (V-1), limonene (V-4), γ-terpinene (V-5), citronellal (V-6), *cis*-dihydrocarvone (V-8), *α*-pinene (V-11), *β*-pinene (V-12), camphene (V-13), sabinene (V-14), *δ*-3-carene (V-15), *p*-cymene (V-19), *α*-humulene(V-23), *δ*-cadinene (V-29), *β*-caryophyllene (V-30), linalool (V-39), citronellol (V-40), geraniol (V-41), *E*-nerolidol (V-43), *α*- terpineol (V-48), borneol (V-51), *α*-bisabolol (V-55), guaiol (V-58), *β*-eudesmol (V-62), citral (V-64), citronellal (V-65), *p*-anisaldehyde (V-72), cuminaldehyde (V-73), *cis*-dihydrocarvone (V-80), carvone (V-82), fenchone (V-85), camphor (V-86), *trans*-anethole (V-90), estragole (V-91), thymol (V-92), eugenol (V-94), elemicin (V-97), dillapiole (V-98), *cis*-limonene oxide (V-102), caryophyllene oxide (V-105), cineole (V-106), *E*-limonene-1,2-oxide (V-107), geranyl acetate (V-109), neryl acetate (V-110), and *n*-hexadecanoic acid (V-118). They were found to exhibit a wide spectrum of antiparasitic activities against human, fish, animal, plants, stored food parasites, and miscellaneous groups. For example, compounds were reported for their antitrypanosomal (17 compounds), antischistosomal (four compounds), antimalarial (13 compounds), antileishmanial (22 compounds), anti-taxoplasmosis (two compounds) activities. Also, compounds were reported for their activities against parasites of stored grains (28 compounds), parasites of plants (18 compounds), parasites of animals (cattle and sheep) (11 compounds) and fish parasitic activity (nine compounds). In addition, several compounds were reported for their activities against: *Musca domestica* (housefly) (25 compounds), *Culex pipiens* (house mosquito) (eight compounds), *Pediculus humanus capitis* (five compounds) (Fig. 3).

## Virtual screening study

The reviewed compounds were analyzed using a virtual screening approach on the PASS (www.way2drug.com) platform, which incorporates sophisticated machine learning algorithms to predict the biological activities of specific structures. This analysis produced scores and a list of predicted biological activities, which were scrutinized for antiparasitic properties, including specific anti-leishmanial, anthelmintic, anti-trypanosomal, anti-coccidial, anti-plasmodial, acaricidal and insecticide activities. The scores and their associated antiparasitic activities were cataloged in an Excel spreadsheet (Table S3) for subsequent statistical evaluation.

**Fig. 4:**
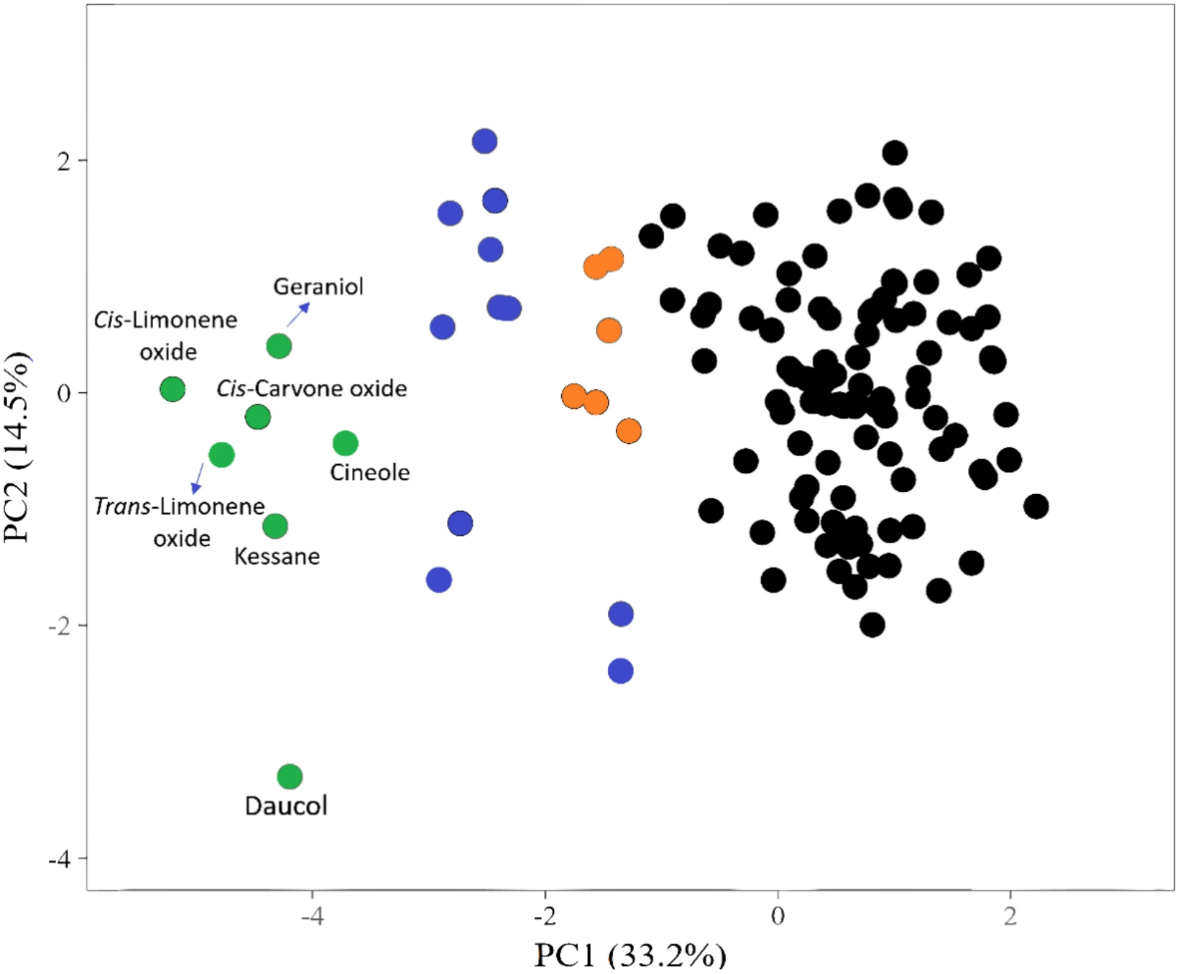
PCA of the Pa scores of the collected terpenes in this review. Black-colored dots represent structures that got scores < 0.5. Orange-colored dots represent structures with scores between 0.5 and 0.6 against one or two parasites. Blue-colored dots represent structures that got scores between 0.5 and 0.6 against three parasites. Green-colored dots represent structures that have scores between 0.5 and 0.6 or higher against more than three parasites.

**Fig. 5:**
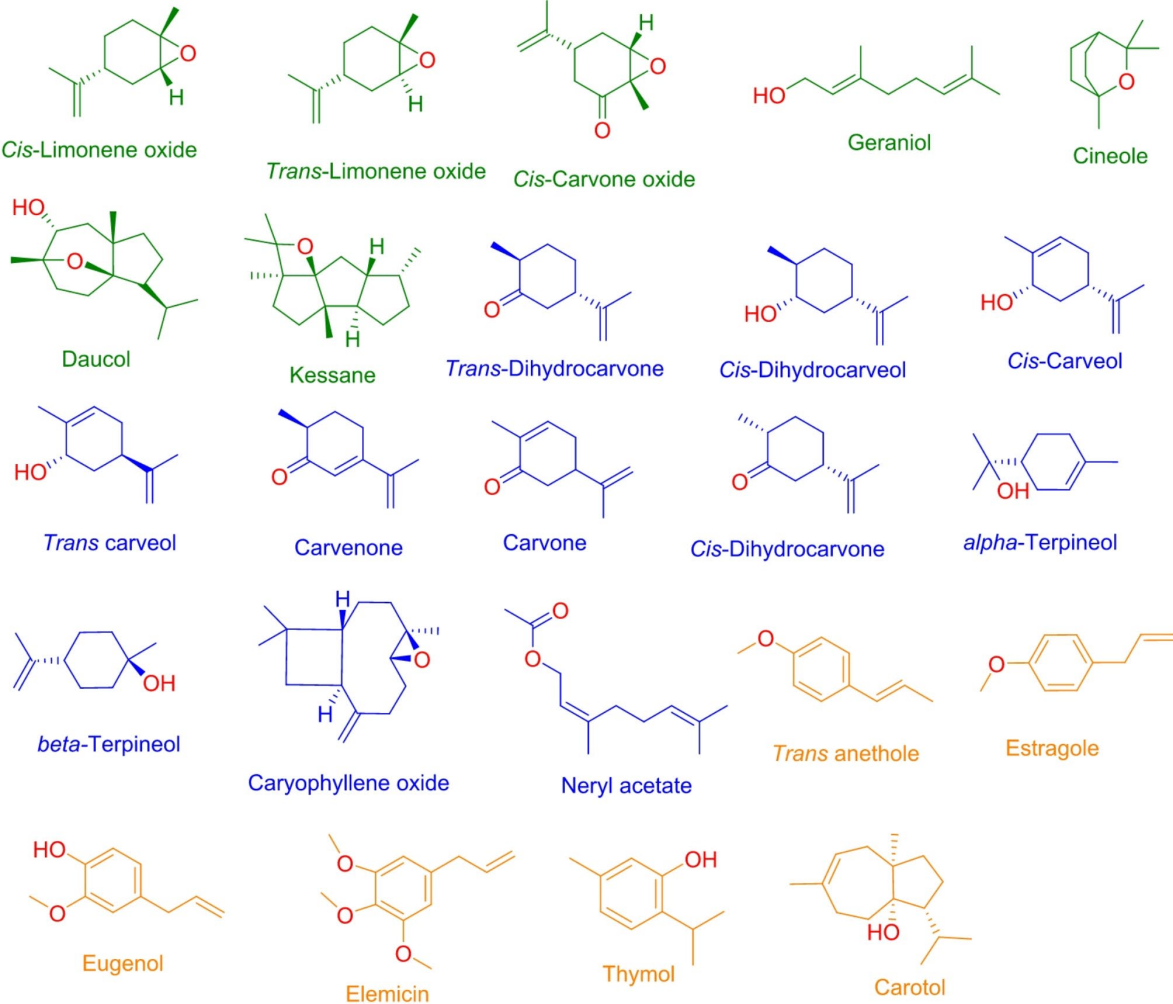
The structures of the top-scoring compounds that were predicted to have antiparasitic activity. Green-colored structures got Pa scores between 0.5 and 0.6 or higher against more than three parasites. Blue-colored structures showed scores between 0.5 and 0.6 against three parasites. Orange-colored structures exhibited scores between 0.5 and 0.6 against one or two parasites.

To differentiate these compounds based on their scores and the range of predicted antiparasitic activities, the data underwent Principle Component Analysis (PCA) based clustering. As depicted in Figs. 4 and 5, most of the screened compounds displayed low predicted antiparasitic activities (i.e., Pa scores below 0.5) and were grouped together, represented as black-colored dots in the analysis. Conversely, 24 oxygenated terpenes exhibited probable activity against at least one parasite, achieving Pa scores of 0.5 or higher.

Among these potentially active compounds, six were identified with Pa scores ranging from 0.5 to 0.6, displaying activity against only one or two parasites, as indicated by orange-colored dots and their structures in Figs. 4 and 5.

The second category of potentially active compounds comprised 11 substances with Pa scores ranging from 0.5 to 0.6, which showed activity against three parasites, as indicated by blue-colored dots and structures. The third category included seven compounds with Pa scores from 0.5 to 0.6 or higher, effective against more than three parasites, depicted as green-colored dots and structures.

These compounds, represented as green in Fig. 5, were then subjected to further analysis to uncover their potential mechanisms of action through inverse docking-based screening.

This investigation was conducted using the PharmMapper virtual screening platform (https://www.lilab-ecust.cn/pharmmapper/), which facilitates rapid and accurate docking of structures against a comprehensive database of protein structures from the RCSB PDB. The affinity scores, or FitScores, for each structure were arranged in an Excel sheet from the highest to the lowest.

Following this, scores and protein interactions specifically related to insects or parasites were manually selected, filtering out protein hits with FitScores above 7. Notably, *Trypanosoma brucei* target pteridine reductase-1 (PTR1, PDB ID: 4WCF) and *Drosophila melanogaster* acetylcholinesterase (AChE; PDB ID: 6XYY) emerged as optimal targets for daucol and geraniol, respectively (with FitScores of 10.23 and 9.49, respectively). Re-docking of these compounds into the active sites of each enzyme revealed that daucol achieved good docking scores of − 7.16 and − 5.02 kcal/mol with PTR1 and AChE, respectively, while geraniol showed scores of − 4.33 kcal/mol and − 7.85 kcal/mol. The interaction of geraniol with AChE was notably comparable to that of a co-crystallized inhibitor, with a docking score of − 7.97 kcal/mol.

Further analysis involved 100-ns long molecular dynamics (MD) simulations to assess the binding stability of these compounds with their target proteins. Daucol demonstrated significant instability, detaching rapidly from the binding sites of both enzymes at early stages of the simulation (at 5.76 and 9.67 ns for PTR1 and AChE, respectively). The same results were shown for geraniol with PTR1, where its modeled structure was detached from the active site of the enzyme at 12.22 ns. These results might be attributed to the small molecular sizes of both structures and lacking multiple stabilizing hydrophilic functional groups, and hence, their calculated absolute binding free energy (Δ*G*_Bind_) were − 2.66 and − 1.67 kcal/mol for daucol with PTR1 and AChE, respectively, and − 2.78 kcal/mol for geraniol with AChE.

**Fig. 6:**
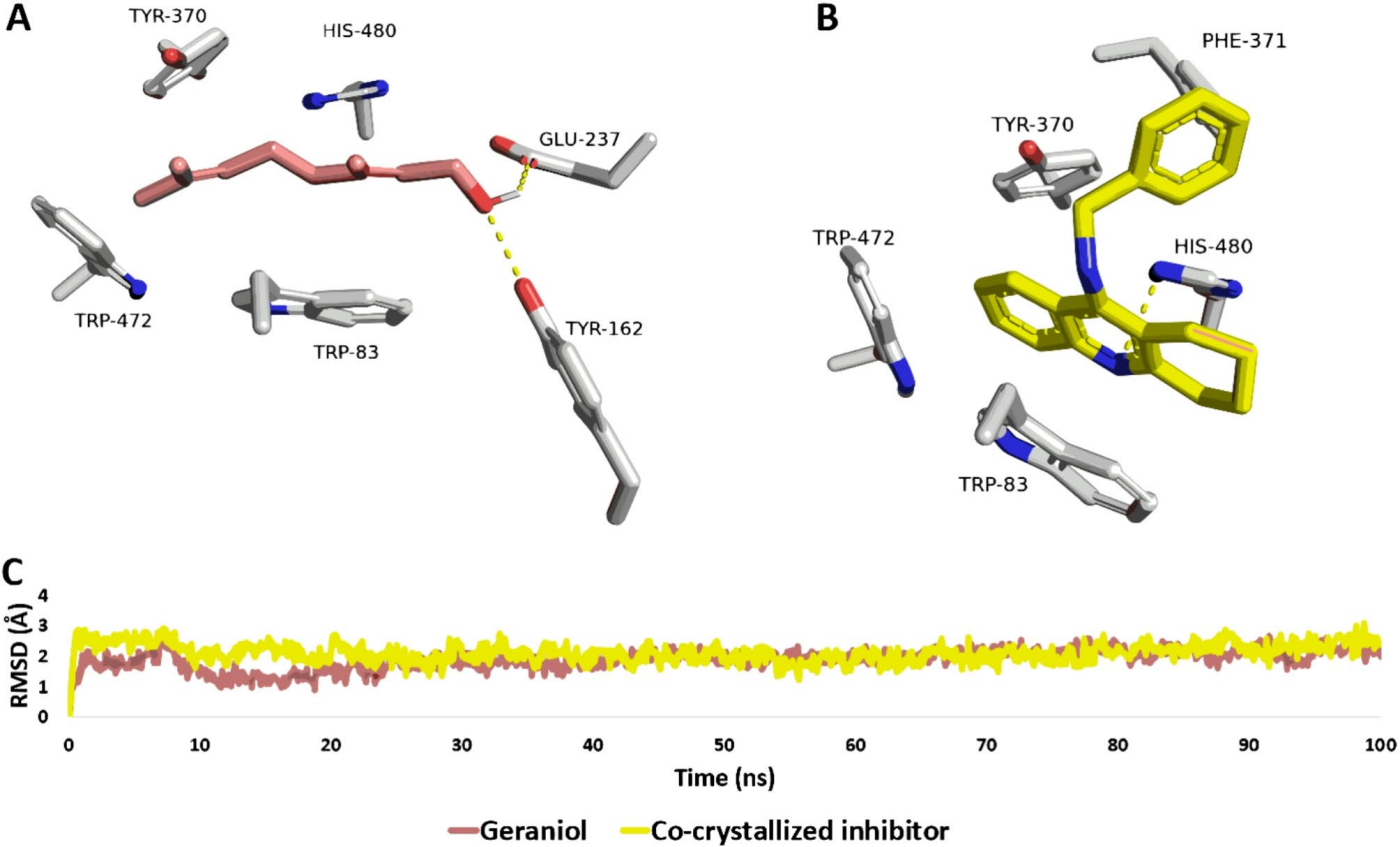
Binding modes of geraniol alongside the co-crystallized inhibitor (tacrine) inside the active site of AChE (PDB ID: 6XYY; **A**,**B**). RMSDs of geraniol, in comparison with tacrine over a 100-ns long MD simulation (**C**).

Conversely, geraniol showed significant binding stability inside the binding tunnel of AChE over a 100-ns long MD simulation, which was comparable to that of the co-crystallized inhibitor (i.e., tacrine). As shown in Fig. 6, the modelled structure of geraniol was able to stabilize its binding inside the binding tunnel of AChE by the establishment of four hydrophobic interactions with TRP-80, TYR-370, TRP-472, and HIS-480 via its hydrophobic hydrocarbon structure. Additionally, it formed strong and durable H-bonds with both TYR-162 and GLU-237 via its terminal hydroxy group. Similarly, tacrine, as the co-crystallized inhibitor, was able to establish stable hydrophobic interactions with the same amino acid residues, while it formed a single stable H-bond with HIS-480. Ultimately, the calculated Δ*G*_Bind_ values for both compounds were closely aligned (-7.88, and − 8.15 kcal/mol, respectively), suggesting that geraniol could be an effective AChE inhibitor and thus a potential broad-spectrum insecticide. Previous studies had also indicated that geraniol is a moderate to potent inhibitor of various AChE variants, supporting its potential utility in this capacity.

Despite the promising potential of in silico studies in identifying novel therapeutic candidates, their translation to in vivo outcomes remains challenging. Computational predictions often fail to account for the complex biological environment, including factors such as bioavailability, metabolism, and systemic toxicity. Moreover, the efficacy observed in virtual models may not always align with real-world biological systems due to differences in target accessibility and compound stability. These limitations underscore the importance of integrating in silico findings with robust in vitro and in vivo validation. By bridging these gaps, this study aims to provide a more comprehensive evaluation of geraniol’s acaricidal potential, addressing both computational and experimental perspectives. For this reason, it was selected for further experimental validation against* S*.* scabeii*^43–46^.

## Scabicidal activity of geraniol

### Materials and methods

#### Geraniol

Geraniol (98%) was purchased from Sigma-Aldrich, Co. (Germany).

#### Scabicidal activity testing

##### In vitro testing^47^

A total of 200 individuals from adult active mite *S*.* scabeii* were collected by skin scrabbing from experimentally infected rabbits, which used for this study. The collected *S. scabeii* mites were transferred to 20 petri dishes (ten *S. scabeii* in each petri dish) using featherweight forceps and micropipette under the dissecting microscope. For each concentration, four petri dishes were used, one of them for the control containing paraffin oil and three replicates of each concentration, the used concentrations from geraniol were 100%, 50%, 25%, 12.5%, and 6.25%, the paraffin oil was used for dilution. Mortality rates were recorded. Data were collected and prepared by using Excel Spreadsheet program (2016). IBM SPSS Statistics (IBM Corp., Armonk, NY, USA) was used to conduct statistical analyses including probit analysis for LC_50_ and LT_50_ determination and the Abbot (1925) calculation for mortality correlation, to evaluate toxicity levels against *S. scabeii* mites. The confidence intervals for the values were 95%.

##### In vivo testing^19,48^

In this study, 20 male New Zeland albino rabbits Dutch strain albino rabbits were used. All rabbits aged six months, weighed 1 to 1.2 kg and free of any disease problem. Five rabbits were kept free from experimental infection with *S. scabeii* mite and considered as negative control group (not infected not treated). Fifteen (15) rabbits were experimentally infected with *S. scabeii* mite and were divided into three groups (5 rabbits/each) as follows: positive control (infected and not treated), positive control (infected and not treated), negative control (not infected not treated), geraniol-treated (paraffin oil-diluted geraniol was applied gently to the infected regions in rabbits day by day), and ivermectin-treated (commercial ivermectin − 1% *w*/*v*, AVICO, Jordan- was subcutaneously injected at a dose of 0.3 mg/kg body weight/rabbit). All rabbits were examined clinically to reveal the improvement along the study period, which lasted for 2 weeks till complete recovery of treated group. An intraperitoneal injection of a ketamine-xylazine mixture (90 mg/kg body wt. ketamine and 10 mg/kg body wt. xylazine) was used to anesthetize before wound creation. The rat’s dorsal surface was shaved using an electric fur clipper, and 70% ethanol was sprayed over it to clean the underlying skin. A full-thickness excision wound (88 mm) was created using a sterile surgical blade and scissors under aseptic conditions, followed by topical application of a pain reliever (Buprenorphine, 0.5 mg/kg body wt.) To mitigate the pain threshold and all efforts were made to minimize suffering. By the end of the experiment, all rabbits were euthanatized after an intraperitoneal injection of a ketamine-xylazine mixture, mentioned above. After that, the treated regions were histopathologically examined. All procedures were performed in accordance with the guidelines of Research Ethics Committee, Assiut Veterinary Medicine, Assiut, Egypt. Deraya University Ethical committee approved the in vivo study presented herein. The ethical approval number is 06/2024/0204 (issued on June 13, 2024). All animal-based studies were conducted according to ARRIVE guidelines.

##### Histopathological examination^49^

Fresh specimens from the noses and ears of all tested rabbits were formalin-fixed and processed for histopathological analysis. Hematoxylin and eosin (H&E) staining was applied to 5-µm thick paraffin sections. The microscopic examination was conducted by light microscopy (Olympus CX31, Tokyo, Japan) and photographed using a digital camera (Olympus, Camedia C-5060, Tokyo, Japan) in the Photomicrograph Lab of the Department of Parasitology, Faculty of Veterinary Medicine, Assiut University, Assiut, Egypt.

Firstly, an in vitro study was done to reveal the effect of geraniol on mites at different concentrations to select the most effective and least corrosive concentration for the proceeding in vivo study. As shown in Table 1, the mortality rate was investigated at the different concentrations (100%, 50%, 25%, 12.5%, and 6.25%), in three replicates. Geraniol showed very strong acaricidal activity in its full strength (100% mortality rate from the first minutes). A mite considered dead when it stopped its mobility for 1 min even with continuous stimulation with needle, showing no more reaction. The mortality rate of adult mites decreased gradually by dilution of the oil. At 6.25% concentration the total mortality rate was obtained after 15 min treatment, so it was chosen as the concentration for a further in vivo study, as it was highly effective against mites but may have less -corrosive properties on the skin of the rabbits.

**Table 1:** The mortality rates of *Sarcoptes scabeii* mites exposed to different concentrations of geraniol in each plate within 15 min.

Mortality rate (%)							
Control	15 min	10 min	5 min	3 min	1 min	Plate	Concentration
0%	100%	100%	100%	100%	100%	P1	100%
0%	100%	100%	100%	100%	100%	P2	
0%	100%	100%	100%	100%	100%	P3	
0%	100%	100%	100%	90%	90%	P1	50%
0%	100%	100%	100%	100%	100%	P2	
0%	100%	100%	100%	100%	90%	P3	
0%	100%	100%	100%	80%	80%	P1	25%
0%	100%	100%	100%	100%	90%	P2	
0%	100%	100%	100%	90%	80%	P3	
0%	100%	100%	80%	80%	60%	P1	12.5%
0%	100%	90%	90%	80%	70%	P2	
0%	100%	100%	100%	90%	50%	P3	
0%	100%	100%	70%	70%	50%	P1	6.25%
0%	100%	90%	80%	60%	40%	P2	
0%	100%	90%	80%	70%	60%	P3	

**Fig. 7:**
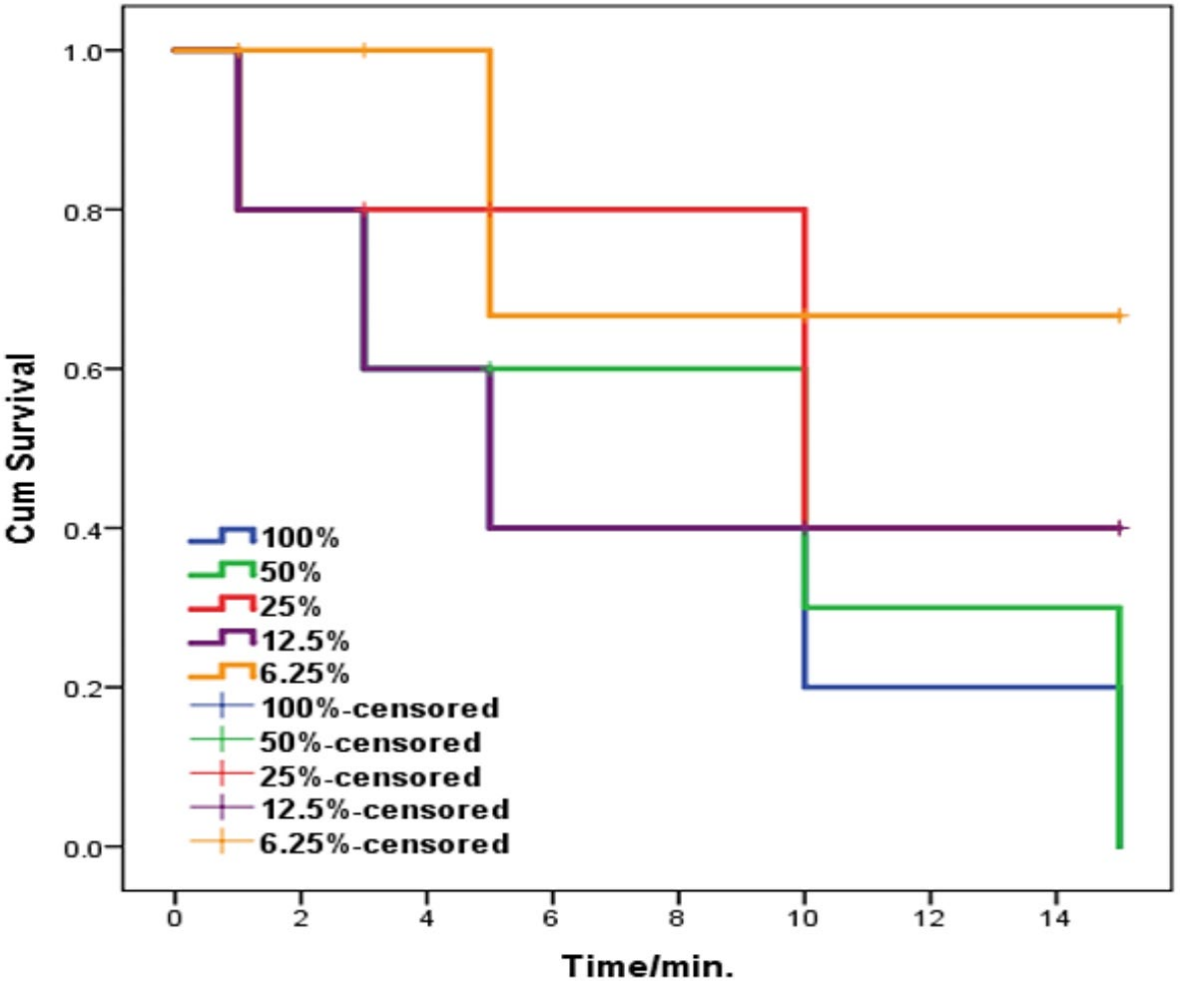
Survival curves of *Sarcoptes scabiei* mites exposed to different geraniol concentrations.

The survival curves (Fig. 7) showed that all concentrations were lethal within 15 min, and exhibiting concentration dependent acaricidal activity, in all concentrations there was significant difference between them and the control group. The average lethal time after exposure to 100%, 50%, 25%, 12.5%, and 6.25% was 1 min, 1.1 min, 1.3 min, 5.4 min., 9.5 min, respectively to all moving mites, while the mites was alive in the control petridish till the end of testing all concentration with 100% survival rate. The LT_50_ values are shown in Table 2.

**Table 2:** Probit regression of the toxicity (LT_50_) of geraniol against *Sarcoptes* mites in vitro.

Compound	LT_50_ (95% CL*)				
	100%	50%	25%	12.5%	6.25%
Geraniol	1 min	1.1 min (1–1.4)	1.3 min (1.1–1.5)	5.4 min (2.6–6.7)	9.5 min (3.2–14.1)

* 95% Confidence Limits.

The LC_50_ value (%) was 0.54 and the LC_90_ value (%) was 0.67, representing very strong miticidal activities (Table 3). The negative control in paraffin oil showing no miticidal activity against adult *S. scabiei* mites, all the adults were still alive for the following 24 h.

**Table 3:** Concentrations of geraniol required to kill 50% (LC_50_) and 90% (LC_90_) of *Sarcoptes* mites at 15 min after exposure.

Compound	LC_50_ (%)	95% CI	LC_90_ (%)	95% CI
Geraniol	0.54	0.45–0.62	0.67	0.61–0.74

### Results

#### Clinical observations

**Fig. 8:**
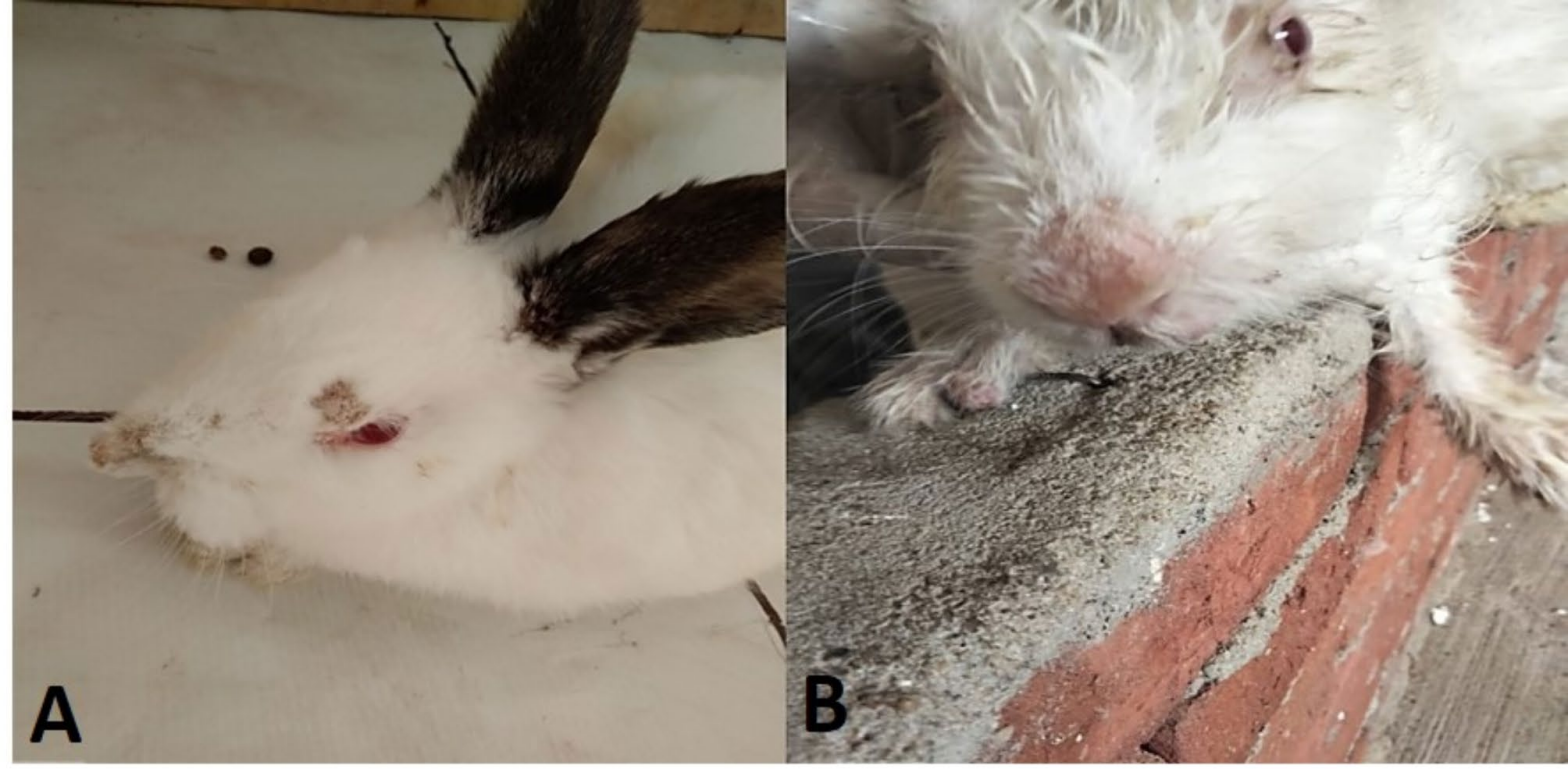
A Rabbit infested with *Sarcoptes scabeii* mite (from the positive control group) showing severe crust formation in the face and recumbency (**A**); a rabbit from the treated group, showing normal behavior and disappearance of crusts from the nose and the face (**B**).

The untreated control rabbits exhibited signs of active *S. scabeii* mite (restlessness, scratching, and multifocal alopecia containing hard dried crusts and scars spread on external ears, nose, face, eyelid, and lips). Throughout the study, two out of the five rabbits (40%) developed complete recumbency and dullness in the last week of the study period (Fig. 8), one rabbit was dead 2 weeks after the start of the study.

**Fig. 9:**
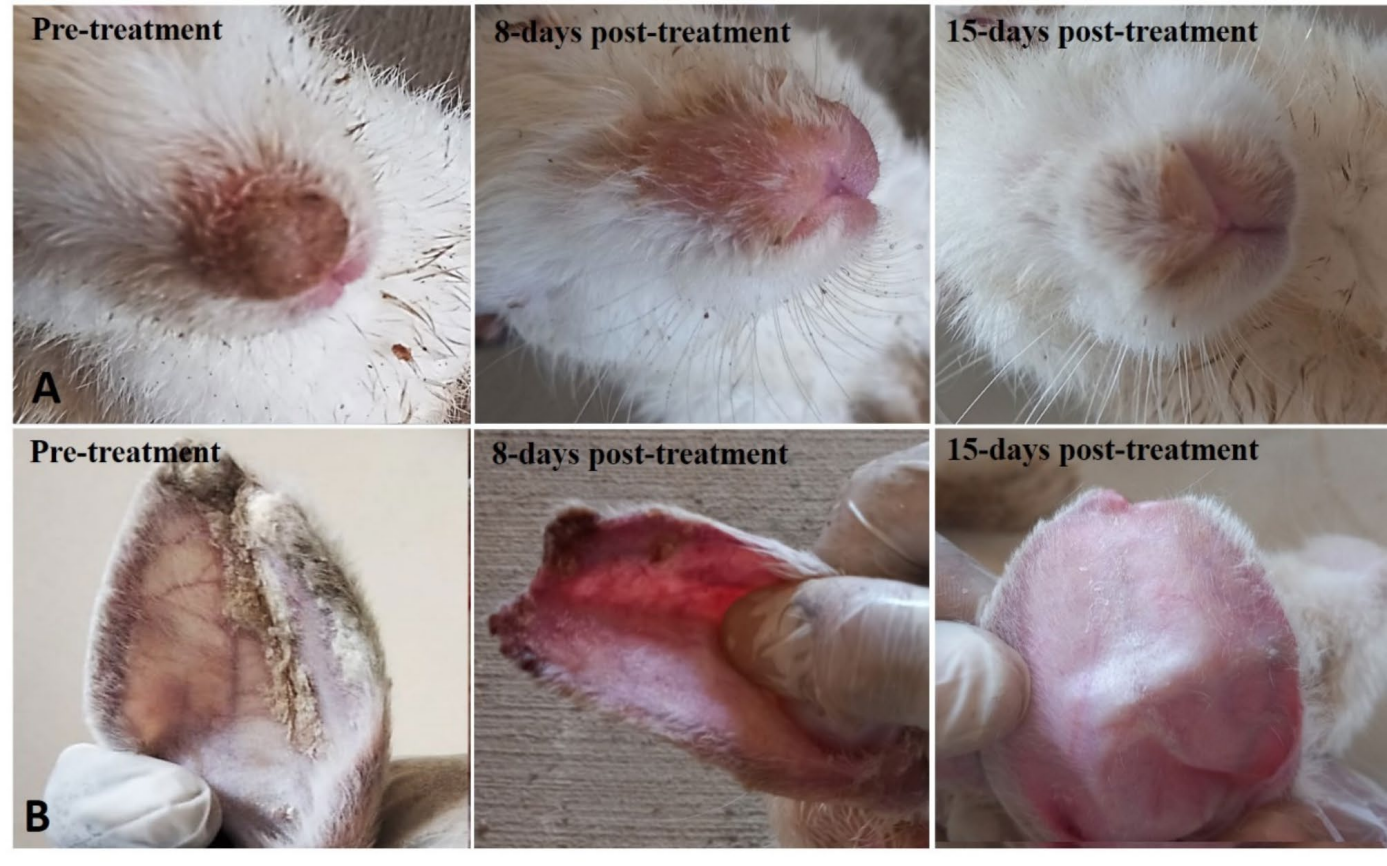
The progressive curative changes in the nose (**A**) and external ear (**B**) of a New Zealand albino rabbit infested with concurrent *Sarcoptes scabeii* mite after treatment with geraniol (6.25%).

The geraniol-treated group expressed complete recovery from clinical mange at 2 weeks post-treatment. This recovery was expressed by the gradual absence of scratching and itching, crusting, restlessness, and skin thickening (Table 4) in addition to the improvement of the general condition of the animals—the treated rabbits exhibited no recumbency and completely normal behavior and activity, in a sharp contrast to the rabbits of the positive control group which showed signs of dullness, recumbency, and general illness. The gross lesions prominently regressed. Smoother skin with new hair growth was observed within the first week following treatment. Complete gross recovery developed at the end of the experiment (2 weeks post-treatment), as a complete absence of crusts, sound skin, and hair growth was observed (Fig. 9). The ivermectin-treated rabbits didn’t develop any clinical healing to the affected skin areas within the experimental period.

**Table 4:** The clinical improvement of skin of rabbits considering crusts and scabs according to Dawod et al.^50^.

Score	0 (No lesion)	1 (Few crusts around ear auricle)	2 (Moderate crusts around ear auricle and nasal skin)	3 (severe crusts around ear auricle and nasal skin)
Negative control	√			
Positive control			√	√
Geraniol treated	√			
Ivermectin treated			√

#### Histopathological observations

**Fig. 10:**
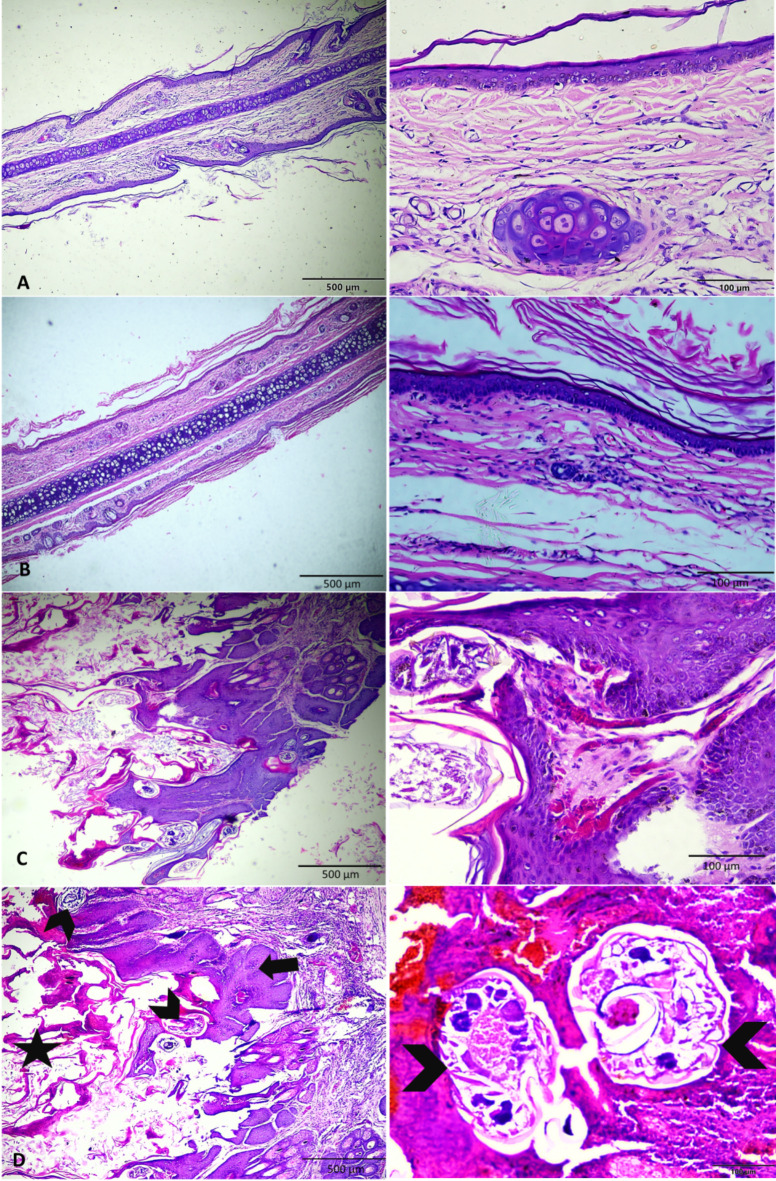
Photomicrographs stained with H&E, showing the histopathology of the ear tissue in white New Zealand rabbits (2 weeks post-treatment). (**A**) Normal appearance of the ear tissue in the rabbit of the − ve control group. (**B**) Regeneration of the epidermal layer, nearly normal dermis, degenerated parasite remnant within the adnexa, slight dermal edema, well-developed fibrous tissue with minimal inflammatory cell infiltration, and mild hyperemia in the geraniol-treated group. (**C**,**D**): Hyperkeratosis (arrow), marked acanthosis with formation of rete ridges, hemorrhage, diffuse inflammatory cells infiltration (neutrophils and eosinophils) throughout the dermis, scabs, presence of mites (chevrons) burrowing the epidermis, and sloughed necrotic epithelial debris on tissue surface (star*) in the + ve control and ivermectin-treated group.

After 2 weeks, the geraniol-treated group showed a marked regeneration in the skin histology and apparent enhancement in its epidermis and dermis with few infiltrations of inflammatory cells, mild edema, and absence of mites (Fig. 10A,B) & (Table 5).

**Table 5:** The clinical improvement of skin of rabbits considering absence of mite and relief of skin in histopathology.

Score	0 (No mite No inflammatory cells No hyperkeratosis	1 (no mite Presence of inflammation) No hyperkeratosis	2 (few mites Presence of inflammation and hyper keratosis	3 (many mites High inflammatory reaction and hyperkeratosis)
Negative control	√			
Positive control				√
Geraniol treated		√		
Ivermectin treated			√	

The positive control group and ivermectin-treated rabbits showed the mite developmental stages *S. scabeii* mite implanted within the epidermal hyperkeratotic layer. Also, the parasite was found intermixed with ulcerated necrotic debris and inflammatory cells on the skin surface and it occasionally made its way deeper into the underlying tissues of the ear. The hyperplasia of the adjacent epidermal cells with marked acanthosis, extensive subepithelial inflammatory cell accumulation and dermal edema, congestion, thrombosis, and extravasation were observed (Fig. 10C,D).

### Discussion

Several essential oils have demonstrated promising result when used against *S*. *scabiei* mites, however, until now not applied commercially, because the commercial manufacturing of these products requires determining the efficacy of each component of the respective essential oils, individually or in combination. These natural substances have been suggested as an alternative to conventional acaricides because of their low toxicity, repellent properties, hindering capacity to the activities of many insect species and their low probability to develop resistance^47,51–53^.

In the present work, geraniol showed a strong acaricidal activity, with a very high mortality rate reaching 100% within 15 min at a concentration of 6.25%. The geraniol-treated rabbits showed obvious clinical and histological improvement in skin lesions, expressed by the absence of crusts and mite stages and tissue regeneration. Geraniol, which is a widely used fragrance monoterpene alcohol found in citronella, rose, and palmarosa oils, has a wide spectrum of pharmacological properties, including antitumor, anti-inflammatory, antioxidative, and antimicrobial activities, as well as hepatoprotective, cardioprotective, and neuroprotective effects^28^. Geraniol had previously been tested in numerous earlier studies against different mite species (*Dermanyssus gallinae* and *Psoroptes cuniculi*), ticks, and head lice^23,29–31^. Concerning *Sarcoptes scabiei*, geraniol at concentration of 1% killed all mites in 24 min; these results are consistent with our findings ^23^. These results agreed with those obtained by Buitrago et al.^54^. Sparagano et al.^55^ demonstrated acaricidal activity of undiluted geraniol against *Dermanyssus gallinae* (100% mortality). However, when it is used at a 10% concentration, mortality decreased to 20.7%. This behavior was associated with a higher concentration of the geraniol exerting a greater acaricidal effect. This is presumably attributed to the various biological properties of geraniol as a volatile monoterpene damaging the membrane and the ion channels of the parasite, altering the action of the membrane-bound proteins and the intracellular signaling cascades^56,57^. The significant lethality that geraniol has demonstrated, even at low concentrations, may be related to the modes of action that it exhibits and the presence of OH group in its structure^58^. The structural OH group affects the binding activity to the neuronal receptors of the insect.

Geraniol, like other naturally occurring volatile oils, may possess neurotoxic properties that impact the central nervous system of the insect. Three main mechanisms were reported for the neurotoxic activity of the essential oils in insects: inhibition of acetylcholinesterase (AChE), antagonism with the biogenic amine messengers octopamine and tryptamine or by affecting gamma-aminobutyric acid (GABA) that acts on the chloride channel^59,60^.

AChE is a key enzyme in the nervous system, regulating acetylcholine levels by catalyzing its hydrolysis across various species. Its inhibition leads to insect death, making it a primary target for insecticides like carbamates and organophosphates. However, their use has declined due to human toxicity and insect resistance^61,62^.

Several volatile oils and their components have demonstrated AChE inhibition activity, though many are weak inhibitors with IC50 values in the mM range (e.g., anisaldehyde, camphor, carvacrol, linalool, menthol, and limonene)^60,63^. Volatile oils from *Cinnamomum cassia* and *Cinnamomum camphora* showed significant acaricidal activity against *Haemaphysalis longicornis* and AChE inhibition, but their major component, trans-cinnamaldehyde, lacked significant AChE inhibition and instead inhibited catalase^64^. Conversely, tea tree oil exhibited strong AChE inhibition against *Pediculus humanus capitis* (IC50: 0.05–0.11 µL/mL), primarily due to 1,8-cineole (IC50: 0.04 µL/mL) and terpinen-4-ol (IC50: 10.3 µL/mL)^65^.

In this study the anti- scabies activity of geraniol is attributed mainly to its AChE inhibitory activity as proven from docking study. However, the activity can involve other mechanisms. For example, anti-inflammatory and antioxidants activity. Recently, mandarin peel oil was reported for its scabicidal activity in rabbits. This activity is due to the downregulation of the immune and inflammatory genes and the upregulation of the anti-inflammatory genes as indicated from mRNA gene expression analysis. Among the detected compounds of mandarin’s oil, geraniol was expected to be potentially responsible for the scabicidal activity of Mandarin oil. Geraniol shows strong binding with glutathione-*S*-transferase (GST) and proinflammatory cytokine interleukin 6 (IL-6) proteins as shown from docking study^66^.

Geraniol can be considered a promising molecule for treating scabies. Its effect both in vitro and in vivo studies is comparable to the commercially available antiscabietics ivermectin and permethrin. Permethrin is a Pyrethroids that acts as GABA-gated chloride channel antagonists. It is usually applied topically (5%) in a single dose. On the other hand, ivermectin is a macrocyclic lactone that acts as GABA agonists, and it binds to glutamate-gated chloride channels in nerve and muscle cells of insects. It is administered orally (200 μg/kg of body weight) as 2 doses over 2-week intervals. Both drugs are relatively safe, although ivermectin is not safe for elderly people, children, pregnant and lactating women and does not have ovicidical activity. Both drugs are reported to develop resistance, as well as to environmental toxicity issues^67–71^. On the other hand, Geraniol has the advantages of being safe for human with no toxicity to environment^72^.

Some herbal medicine formulations with significant anti-scabies activity were described^73,74^. However, geraniol is a volatile compound, so its effectiveness can be reduced over time. This challenge can be solved by preparation of dosage forms that enhance its efficiency. For example, geraniol incorporated polymer disks or pellets were prepared and found to be effective against *Aedes albopictus*. Another example is the preparation of encapsulated polymeric micelles of geraniol and other monoterpenes. This formulation was found to be stable after twelve months and significant pediculicidal activity against head lice^75^. Additionally, several nanoparticle formulations containing geraniol were reported for their insecticidal activity either in plants or animals^76–78^.

The limitations observed during in vivo testing of geraniol include the absence of data addressing potential side effects or the varying impacts on different skin types. While geraniol demonstrated promising acaricidal activity and skin regeneration without adverse effects in rabbits, the manuscript does not explore its tolerability across a broader spectrum of skin sensitivities or its potential for irritation, especially in cases of prolonged use. Furthermore, the volatility of geraniol poses a challenge for maintaining efficacy, emphasizing the need for stable formulations. Addressing these gaps through human clinical trials and detailed dermatological assessments would provide a more comprehensive understanding of its clinical potential​.

## Conclusions and future perspectives

This study has explored the potential of geraniol, a naturally occurring monoterpene alcohol, as an acaricidal agent against *S*.* scabiei*, the mite responsible for scabies. Our findings demonstrate that geraniol exhibits promising acaricidal activity: In vitro studies revealed high mortality rates of *S. scabiei* mites at geraniol concentrations as low as 6.25%. In vivo experiments on rabbits infected with *S. scabiei* showed significant clinical and histological improvement in geraniol-treated animals compared to the control groups. These results warrant further investigations with geraniol as a potential therapeutic option for scabies treatment.

Based on these promising findings, future research should explore several key areas:

Formulation development: Developing a user-friendly and effective topical formulation containing geraniol is crucial for practical application. Factors like concentration, penetration enhancers, and stability need to be optimized. Given its volatile nature, maintaining efficacy over time requires innovative delivery systems, such as encapsulation in polymeric micelles or nanoparticles, to enhance stability and usability.

Mechanism of action: Elucidating the precise mechanisms by which geraniol exerts its acaricidal effect against *S. scabiei* would provide valuable insight for the optimization of its use and for potential combination therapies.

Combination therapy: Investigating potential synergies between geraniol and other acaricides could enhance efficacy and might possibly reduce the risk of resistance development in mite populations.

Clinical trials: Conducting well-designed clinical trials is essential to assess the safety and efficacy of geraniol-based formulations for scabies treatment in humans. This would involve determining optimal dosage, treatment duration, and minimizing possible side effects.

Resistance monitoring: Long-term monitoring of *S. scabiei* populations for potential development of resistance to geraniol is crucial for ensuring the sustainability of this approach. By addressing these future directions, geraniol has the potential to become a valuable addition to the scabies treatment armamentarium, offering a potentially safe, effective, and natural alternative to conventional acaricides.

## Electronic supplementary material

Below is the link to the electronic supplementary material.

Supplementary Material 1

## Data Availability

All data generated or analyzed during this study are included in this published article (and its Supplementary Information files).
