# Supplementary material for: Antiparasitic efficacy of geraniol from Apiaceae family in scabies treatment
Source: Sci Rep. 2025 May 15;15:16928. doi: 10.1038/s41598-025-97702-z (PMC12081939; doi:10.1038/s41598-025-97702-z)
Supplement: Supplementary file 1 — Supplementary Material 1 [file 41598_2025_97702_MOESM1_ESM.pdf]

Supplementary File

Antiparasitic Efficacy of Geraniol from Apiaceae Family in Scabies Treatment

Iman S. A. Khallaf[1*], Lourin G. Malak[2], Soad A. L. Bayoumi[2], Salwa F. Farag[2,3], Ahmed M. Sayed[4,5], Sara A.A. Mohamed[6], Asmaa A. E. Nasr[7], Radwa Y. Ibrahim[8], Eman M. Zahran[9], Gerhard Bringmann[10], Usama Ramadan Abdelmohsen[9,11*]

[1]Pharmacognosy and Natural Products Department, Faculty of Pharmacy, Menoufia University, Shibin Elkom, 32511, Egypt; Iman.Khallaf123@phrm.menofia.edu.eg

[2]Pharmacognosy Department, Faculty of Pharmacy, Assiut University, Assiut 71526, Egypt; lourinmalak@aun.edu.eg; soad.bayoumi@pharm.aun.edu.eg; salwa.sayed@pharm.aun.edu.eg

[3] Pharmacognosy Department, College of Pharmacy, Taif University, P.O. Box 11099, Taif 21944, Saudi Arabia

[4] Department of Pharmacognosy, collage of Pharmacy, Almaaqal University, 61014 Basrah, Iraq; ahmed.mohammed@almaaqal.edu.iq

[5]Department of Pharmacognosy, Faculty of Pharmacy, Nahda University, 62513 Beni-Suef, Egypt.

[6]Department of Parasitology, Faculty of Veterinary Medicine Assiut University, Assiut 71515, Egypt; salma@aun.edu.eg

[7]Department of Poultry Diseases, Animal Health Research Institute, Assiut Regional Laboratory, Agricultural Research Center (ARC), Assiut, Egypt; asmaa.abdelghafar66@vet.aun.edu.eg

[8]Department of Medical Parasitology, Faculty of Medicine, Assiut University, Assiut, Egypt; radwa.yassin@aun.edu.eg

[9]Department of Pharmacognosy, Faculty of Pharmacy, Deraya University, 7 Universities Zone, 61111 New Minia City, Egypt; eman.maher@deraya.edu.eg

[10] Institute of Organic Chemistry, University of Würzburg, Am Hubland, 97074, Würzburg, Germany; gerhard.bringmann@uni-wuerzburg.de

[11]Deraya Center for Scientific Research; Deraya University, 7 Universities Zone, 61111 New Minia City, Egypt; Usama.ramadan@mu.edu.eg

* **Corresponding authors:** Iman.Khallaf123@phrm.menofia.edu.eg; usama.ramadan@mu.edu.eg

Table S1: A list of natural volatile oil components (V-1 - V-122) isolated from different Apiaceae plants.

A list of natural volatile oil components (V-1 - V-122) isolated from different Apiaceae plants.				
No.	Compound name	Structure	Source	Ref.
1- Terpene hydrocarbons<br>A- Monoterpenes hydrocarbons				
V-1	*β*-Myrcene	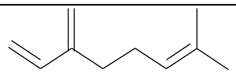	*Anethum graveolens*<br>*Foeniculum vulgare*<br>*Ferula sp.*<br>*(Assafoetida)*<br>*Coriandrum sativum*<br>*Apium graveolens*<br>*Carum carvi*	[1-7]
V-2	(*Z*)-β-Ocimene	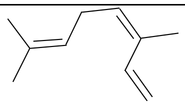	*Ferula assafoetida*<br>*Apium graveolens*	[4, 6]
V-3	(*E*)-β-ocimene	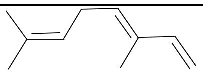	*Ferula assafoetida*	[6]
V-4	Limonene	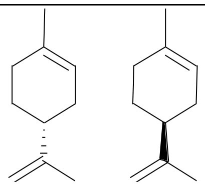	*Foeniculum vulgare*<br>*Coriandrum sativum*<br>*Anethum graveolens*<br>*Carum carvi*<br>*Petroselinum*<br>*crispum*<br>*Apium graveolens*<br>*Ferula sp.*<br>*(F. assafoetida)*	[1-4, 6-9]
V-5	*γ*-Terpinene	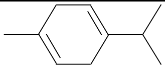	*Foeniculum vulgare,*<br>*Coriandrum sativum*<br>*Anethum graveolens*<br>*Carum carvi*<br>*Apium graveolens,*<br>*Cuminum cyminum*<br>*Ferula assafoetida*	[2, 4, 6-8]
V-6	*α*-Terpinene	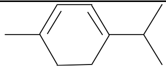	*Ferula assafoetida*	[6]
V-7	Isoterpinolene	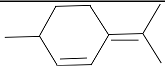	*Ferula assafoetida*	[6]
V-8	Terpinolene	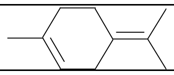	*Ferula assafoetida*	[6]

V-9	α-Phellandrene	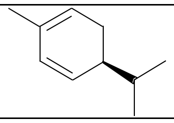	*Anethum graveolens*<br>*Ferula assafoetida*<br>*Angelica glauca*	[2, 6, 10]
V-10	(-)-β-Phellandrene	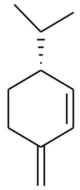	*Ferula assafoetida*<br>*Angelica archangelica*<br>*Anthriscus caucalis M. Bieb*	[6, 11]
V-11	α-Pinene	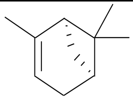	*Foeniculum vulgare*<br>*Anethum graveolens*<br>*Carum carvi*<br>*Coriandrum sativum*<br>*Apium graveolens*<br>*Cuminum cyminum*<br>*Petroselinum crispum Ferula sp. (F. Assafoetida)*<br>*Angelica archangelica*	[1-4, 6-10]
V-12	(+/−)-β-Pinene	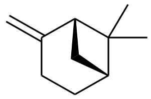 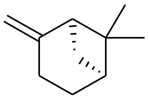	*Coriandrum sativum*<br>*Petroselinum crispum*<br>*Apium graveolens*<br>*Foeniculum vulgare*<br>*Ferula sp (F. Assafoetida)*<br>*Angelica glauca*<br>*Ferula kuhistanica Korovin*	[1-8, 10, 12]
V-13	(+/−) -Camphene	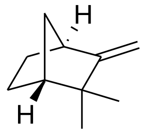	*Foeniculum vulgare*<br>*Coriandrum sativum*<br>*Ferula assafoetida*<br>*Anthriscus caucalis M. Bieb*	[6-8, 11]
V-14	(+/−) -Sabinene	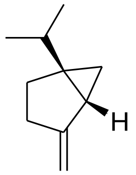 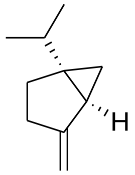	*Foeniculum vulgare*<br>*Coriandrum sativum*<br>*Anethum graveolens*<br>*Carum carvi*<br>*Apium graveolens*<br>*Cuminum cyminum*<br>*Ferula assafoetida*<br>*Ferula kuhistanica Korovin*	[2-4, 6, 8, 12]
V-15	δ-3-Carene	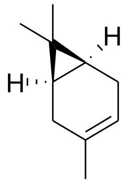	*Angelica archangelica* [35]<br>*Ferula assafoetida*<br>*Seseli tortuosum subsp. tortuosum and S. tortuosum subsp. maritimum*	[6, 13]

V-16	(+,-)-α-Thujene	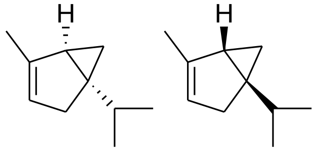	*Ferula assafoetida*	[6, 11]
V-17	α-Fenchene	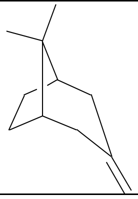	*Ferula assafoetida*	[6]
V-18	Thuja-2,4(10)-diene (Dehydrosabinene)	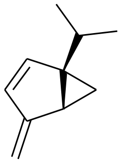	*Ferula assafoetida*	[6]
V-19	*P*-Cymene	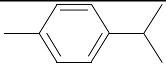	*Foeniculum vulgare*<br>*Coriandrum sativum*<br>*Anethum graveolens,*<br>*Cuminum cyminum*<br>*Carum carvi,*<br>*Petroselinum crispum*<br>[1-5]	[1-3, 7-9]
1- Terpene hydrocarbons<br>b-Sesquiterpene hydrocarbons				
V-20	Germacrene B	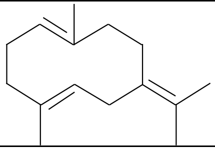	*Ferula sp.* [13]	[5]
V-21	(-)-Germacrene D	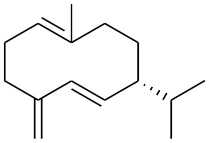	*Ferula sp.* [13]<br>*Anthriscus caucalis M. Bieb.*	[5, 11]
V-22	Germacrene	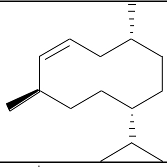	*Coriandrum sativum*	[1]
V-23	α-Humulene	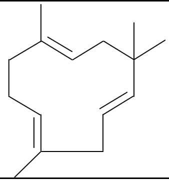	*Apium graveolens*	[4]
V-24	(+/−) Bicyclogermacrene	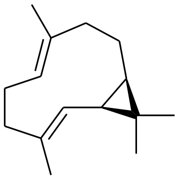	*Eryngium bourgatii* Gouan	[14]

V-25	*γ*-Curcumene	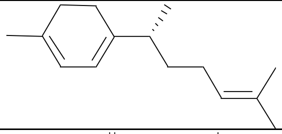	*Apium graveolens* [12]	[4]
V-26	*β*-Bisabolene	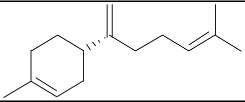	*Daucus carota*<br>*Pimpinella anisum*	[7, 15]
V-27	*trans*-*β*-Guaiene	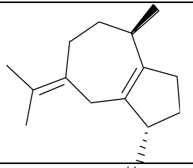	*Apium graveolens*	[4]
V-28	*γ*-Muurolene	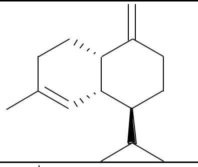	*Eryngium bourgatii* Gouan	[14]
V-29	*δ*-Cadinene	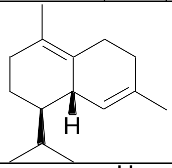	*Ferula sp.*	[5]
V-30	*β*-caryophyllene	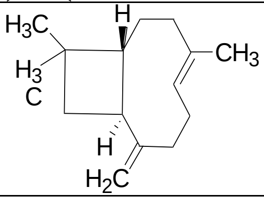	*Ferula sp.*<br>*Coriandrum sativum*	[1, 5]
V-31	*E*-Caryophyllene	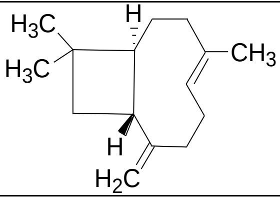	*Eryngium bourgatii* Gouan<br>*Carum carvi*<br>*Apium graveolens*	[4, 7, 14]
V-32	*β*-Selinene	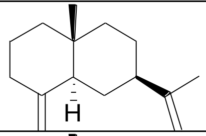	*Sanicula europae*	[14]
V-33	*α*-Selinene	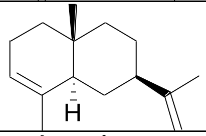	*Sanicula europaea*<br>*Apium graveolens*	[4, 14]
V-34	Valencene	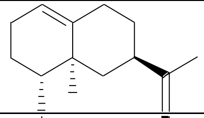	*Eryngium glaciale* Boiss.	[14]
V-35	*α*-Ylangene	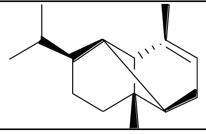	*Ferula assafoetida*	[6]

V-36	*α*-Copaene	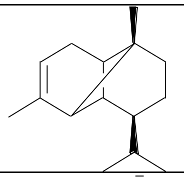	*Ferula assafoetid*	[6]
V-37	*β*-Cubebene	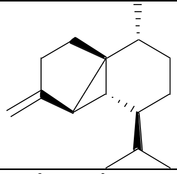	*Ferula assafoeti*	[6]
V-38	Aristolene	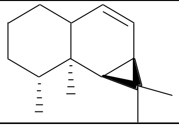	*Ferula assafoetida*	[6]
2- Terpene oxygenated terpene compounds<br>A- Alcohol terpene compounds				
V-39	3S-(+)-Linalool	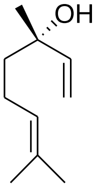	*Coriandrum sativum*<br>*Cuminum cyminum*<br>*Carum carvi*<br>*Ferula sp*<br>*(F. Assafoetida)*	[1, 2, 5, 6, 8, 16]
V-40	(+/−)-Citronellol	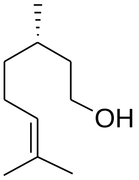<br>(-)	*Coriandrum sativum*	[2]
V-41	Geraniol	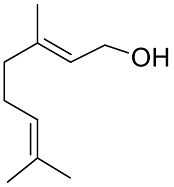	*Coriandrum sativum*	[1, 2]
V-42	(-)-*α*-Santoline alcohol	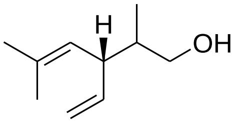	*Apium graveolens*	[4]
V-43	(+,-)-*E*-Nerolidol	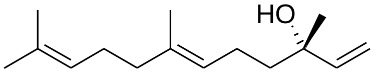<br>(+)<br>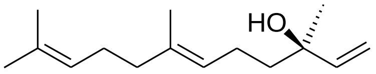<br>(-)	*Eryngium corniculatum* Lam.	[14]

V-44	*cis*-Carveol	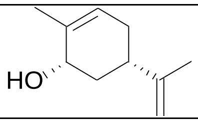	*Anethum graveolens* *Carum carvi*	[8]
V-45	(+,-)-Dihydrocarveol	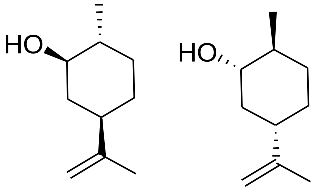 (-) (+)	*Anethum graveolens* *Carum carvi*	[8]
V-46	*neo-iso*-Dihydrocarveol	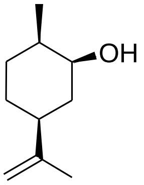	*Carum carvi*	[7]
V-47	*β*-Terpineol	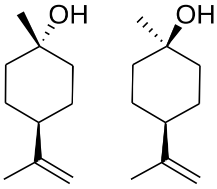	*Cuminum cyminum*	[2]
V-48	(+,-)-*α*-Terpineol	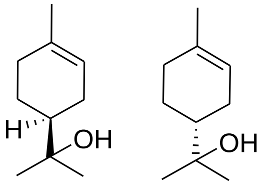 (-) (+)	*Ferula sp* *Coriandrum sativum*	[1, 5]
V-49	*trans*-Carveol	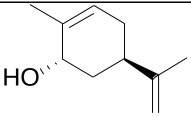	*Angelica glauca* *Carum cavi*	[7, 10]
V-50	(+,-)-*trans*-*p*-Menth-2-en-1-ol	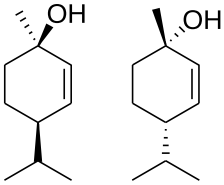	*Ferula assafoetida*	[6]
V-51	(+, -)-Borneol	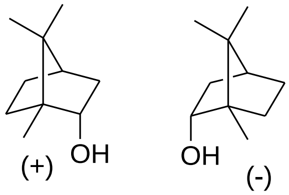 (+) (-)	*Coriandrum sativum*	[1]
V-52	Pinocarveol	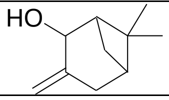	*Cuminum cyminum*	[2]

V-53	*trans*-Sabinene hydrate	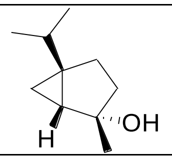	*Ferula assafoetida*	[6]
V-54	*cis*-Sabinene hydrate	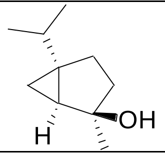	*Ferula assafoetida*	[6]
V-55	*α*-Bisabolol	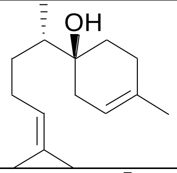	*Eryngium palmatum* Vis. et Pancic	[14]
V-56	Carotol	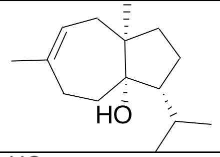	*Cuminum cyminum* *Daucus carota*	[2, 15]
V-57	*α*-Cadinol	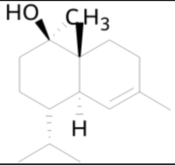	*Ferula sp* *Angelica urumiensis* [13, 35] *Angelica urumiensis*	[5, 10]
V-58	Guaiol	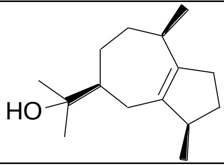	*Ferula sp.*	[5]
V-59	Spathulenol	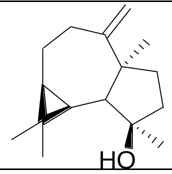	*Ferula sp.*	[5]
V-60	Ylangenol	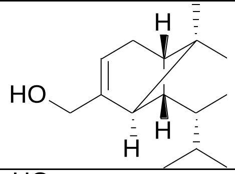	*Daucus carota*	[15]
V-61	Daucol	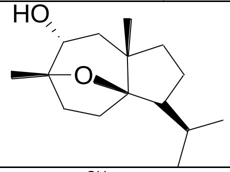	*Daucus carota*	[15]
V-62	β-Eudesmol	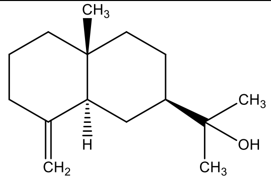	*Eryngium corniculatum* Lam. *Angelica gigas*	[10, 14]

V-63	(+,-)-1-Phenyl-1,2-ethanediol	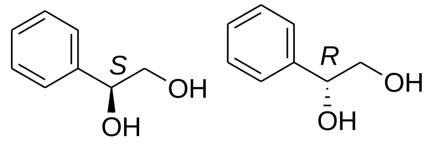	*Apium graveolens*	[4]
2- Terpene oxygenated terpene compounds<br>B) Aldehyde terpene compounds				
V-64	Citral	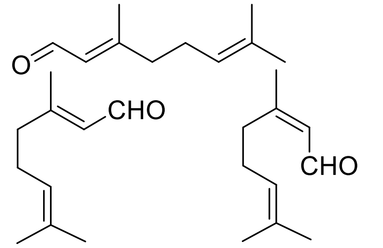	*Coriandrum sativum*	[2]
V-65	Citronellal	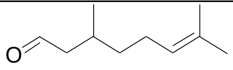	*Ferula assafoetida*	[6]
V-66	Decanal	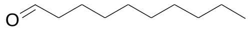	*Ferula assafoetida*	[6]
V-67	(1*R*)-(-)-Myrtenal	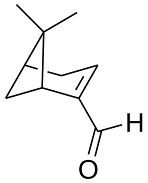	*Cuminum cyminum*	[2]
V-68	(+/-)-α-Campholene-aldehyde	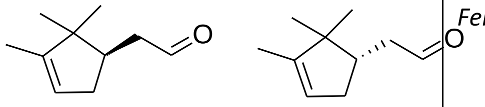<br>(+) (-)	*Ferula assafoetida*	[6]
V-69	α-Thujenal	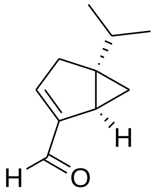	*Cuminum cyminum*	[9]
V-70	(2S)-2-Methyl, 3-phenyl propanal	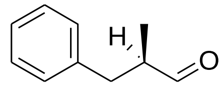	*Cuminum cyminum*	[9]
V-71	Benzaldehyde	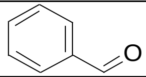	*Foeniculum vulgare*	[9]
V-72	*p*-Anisaldehyde	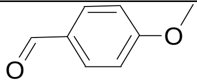	*Foeniculum vulgare*<br>*Pimpinella anisum*	[2, 3, 8]
V-73	Cuminaldehyde	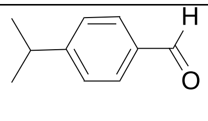	*Cuminum cyminum*<br>*Foeniculum vulgare*	[2, 3]
V-74	2,4,6-Trimethyl-benzaldehyde	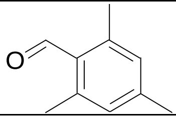	*Eryngium corniculatum* Lam.	[14]

V-75	2,4,5-trimethyl-benzaldehyde	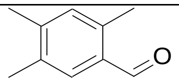	*Eryngium corniculatum* Lam.	[14]
V-76	2,3,6-Trimethyl-benzaldehyde	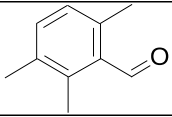	*Eryngium amethystinum*	[14]
V-77	2,3,4-Trimethyl-benzaldehyde	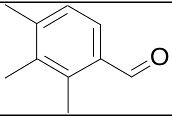	*Eryngium amethystinum*	[14]
3- Terpene oxygenated terpene compounds<br>B- Ketone terpene compounds				
V-78	(6R, 10R)-Hexahydrofarnesyl acetone	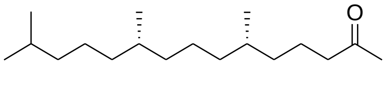	*Angelica urumiensis*	[10]
V-79	(+/-)-Cryptone	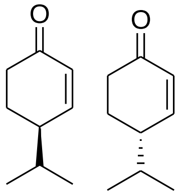	*Eryngium glaciale Boiss*	[14]
V-80	*cis*-Dihydrocarvone	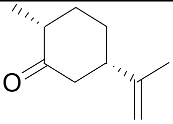	*Carum carvi*<br>*Anethum graveolens*<br>*Pimpinella anisum*	[2, 7-9]
V-81	*trans*-Dihydrocarvone	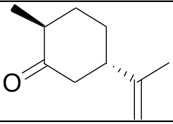	*Anethum graveolens*<br>*Carum carvi*	[2, 7-9]
V-82	*S*-(+)-Carvone	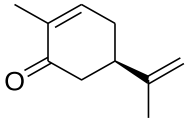	*Anethum graveolens*<br>*Carum carvi*<br>*Coriandrum sativum*<br>*Foeniculum vulgar*	[2, 7-9, 17]
V-83	(+/-)Carvenone	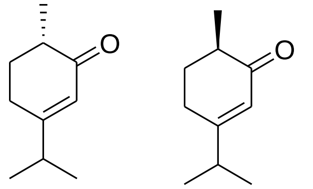<br>(-) (+)	*Carum carvi*	[2]
V-84	Anisketone	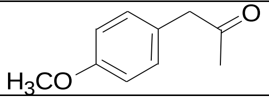	*Foeniculum vulgare*	[3]
V-85	Fenchone	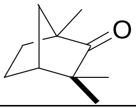	*Foeniculum vulgare*	[2, 8, 9]

V-86	Camphor	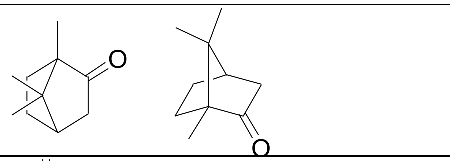	*Coriandrum sativum*<br>*Foeniculum vulgare*	[1, 3, 7, 8]
V-87	Pinocarvone	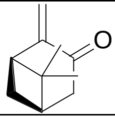	*Ferula assafoetida*	[6]
V-88	6-Camphenone	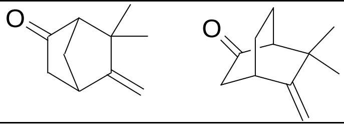	*Eryngium glaciale* Boiss	[14]
V-89	Cyclocolorenone	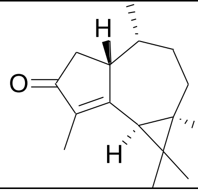	*Sanicula europaea*	[14]
2-Terpene oxygenated terpene compounds<br>D-Phenol and phenolic ethers terpene compounds				
V-90	*trans*-Anethole	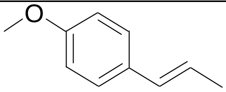	*Foeniculum vulgare*<br>*Pimpinella anisum*<br>*Coriandrum sativu*	[1-3, 7-9]
V-91	Estragole<br>(methyl chavicol)	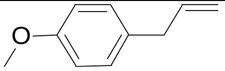	*Foeniculum vulgare*<br>*Pimpinella anisum*<br>*Coriandrum sativum*	[1-3, 7-9]
V-92	Thymol	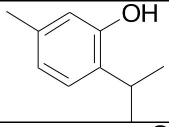	*Carum carvi*<br>*Anethum graveolens*	[9]
V-93	4-Vinylguaiacol	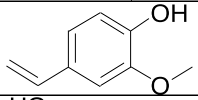	*Petagnaea gussonei* (Sprengel) Rauschert	[14]
V-94	Eugenol	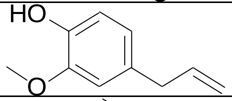	*Pimpinella anisum*	[2]
V-95	Isomyristicin	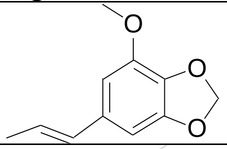	*Anethum graveolens*	[2]
V-96	Myristicin	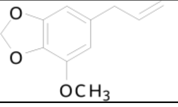	*Anethum graveolens*<br>*Petroselinum crispum*	[2]
V-97	Elemicin	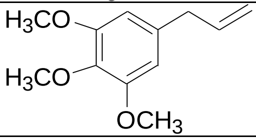	*Petroselinum crispum*	[2]
V-98	Dillapiole	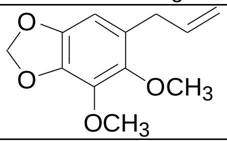	*Anethum graveolens*	[2, 8, 9]

V-99	Apiol	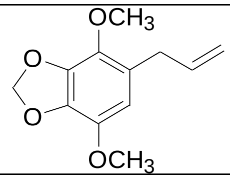	*Petroselinum crispum*	[2]
2-Terpene oxygenated terpene compounds<br>E-Oxides terpene compounds				
V-100	*cis*-Linalool oxide	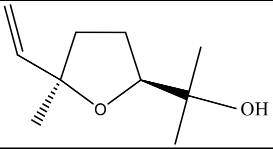	*Coriandrum sativum*	[8]
V-101	*trans-Linalool oxide*	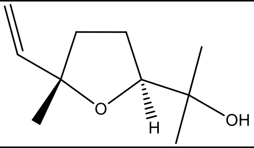	*Coriandrum sativum*	[8]
V-102	*cis*-Limonene oxide	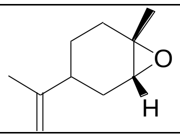	*Anethum graveolens*<br>*Carum carvi*	[7, 8]
V-103	*trans*-Limonene oxide	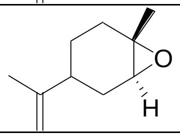	*Carum carvi*	[7, 8]
V-104	*cis*-Carvone oxide	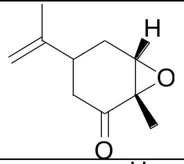	*Carum carvi*	[8]
V-105	Caryophyllene oxide	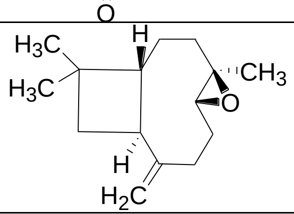	*Carum carvi*<br>*Ferula sp.*<br>*Daucus carota*	[5, 8, 15]
V-106	Cineole	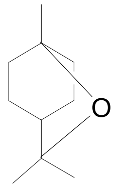	*Anethum graveolens*	[2]
V-107	*E*-Limonene-1,2-oxide	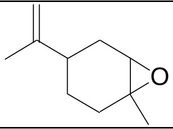	*Carum carvi*	[9]
V-108	Kessane	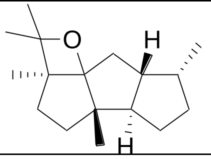	*Apium graveolens*	[4]
2-Terpene oxygenated terpene compounds<br>F-Esters and lactones terpene compounds				

V-109	Geranyl acetate	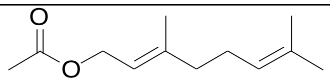	*Coriandrum sativu*	[1, 7]
V-110	Neryl acetate	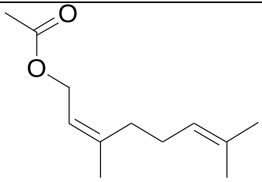	*Ferula sp*	[5]
V-111	Fenchyl acetate	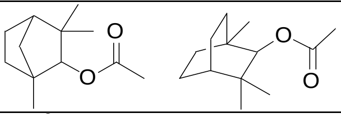	*Ferula assafoetida*	[6]
V-112	Isonicotinic acid, dodecyl ester	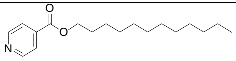	*Daucus carota*	[15]
V-113	*Z*-Ligustilide	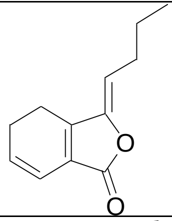	*Angelica glauca*	[10]
V-114	*Z*-Butylidene phthalide	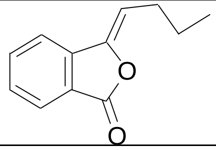	*Angelica glauca*	[10]
2-Terpene oxygenated terpene compounds<br>g-Sulfur terpene compounds				
V-115	*sec*-Butyl-*Z*-propenyl disulfide	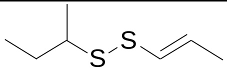	*Ferula sp*<br>*assafoetid*	[5, 6]
V-116	*sec*-Butyl-*E*-propenyl disulfide	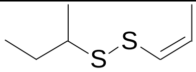	*Ferula sp.*<br>*F. assafoetida*	[5, 6]
3-Miscellaneous compounds				
V-117	*n*-Butyl-1,4-cycloheptadiene	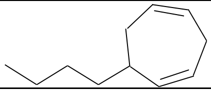	*Apium graveolens*	[4]
V-118	*n*-Hexadecanoic acid	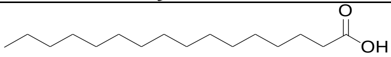	*Daucus carota*<br>*Petagnaea gussonei* (Sprengel) Rauschert	[14, 15]
V-119	*Cis*-Oleic acid	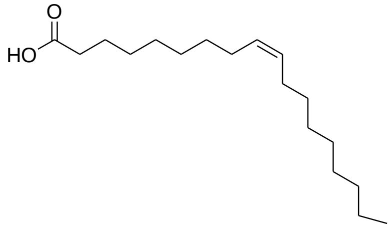	*Daucus carota*	[15]
V-120	2,5-Pyrrolidinedione	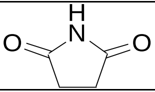	*Apium graveolen*	[4]

V-121	2-(3-propenyl)-furan	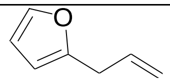	*Apium graveolens*	[4]
V-122	*Z*,*Z*-9,12-Octadecadienoic acid	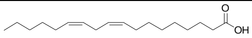	*Petagnaea gussonei* (Sprengel) Rauschert	[14]

Table S2. Antiparasitic activities of the screened compounds.

No.	Compound	Activity [Ref.]
**1. Compounds with anti-trypanosomal activity 2.**		
V-4	**Limonene**	D-(+)-limonene active against epimastigote of *T. cruzi* (IC_{50} 42 ± 2.5 µg/mL) [18, 19][13, 14], *T. cruzi* Tulahuen 2 strain (IC_{50} > 50 µM) [20][15], trypomastigote of *T. cruzi* IC_{50} 9 ± 0.8 µg/mL, amastigote of *T. cruzi* (IC_{50} 29 ± 0.7 µg/mL) [19][14]
V-5	**γ-Terpinene**	*In vitro* antiprotozoal activity against blood stream forms of *T. brucei rhodesiense* STIB 900 strain (IC_{50} 32.9 ± 26 µg/mL), trypomastigote forms of *T. cruzi* (IC_{50} > 90 µg/mL) [21] [16]
V-6	***α*-Terpinene**	*In vitro* activity against blood stream forms of *T. brucei rhodesiense* STIB 900 strain (IC_{50} 3.1 ± 1.6 µg/mL), trypomastigote forms of *T. cruzi* (IC_{50} 49.1 ± 7.3 µg/mL) [21] [16]
V-11	**α-Pinene**	*In vitro* antiprotozoal activity against the *T. cruzi* epimastigotes (IC_{50} 2.74 µg/mL), amastigotes (IC_{50} 1.92 µg/mL) [18][13], blood stream forms of *T. brucei rhodesiense* e STIB 900 strain (IC_{50} 0.42 ± 0.24 µg/mL), trypomastigote forms of *T. cruzi* (IC_{50} > 90 µg/mL) [16] and *T. brucei* (blood stream) TC221/Baltz medium ED_{50} = 4.1 µg/mL [22]
V-13	**Camphene**	*In vitro* activity against *T. cruzi* IC_{50} = 48.0 ± 1.6 µg/mL, *T. brucei* TC221 (IC_{50} 80.66 ± 0.87 µg/mL) [23]
V-19	***p*-Cymene**	*In vitro* activity against blood stream forms of *T. brucei rhodesiense* STIB 900 strain (I_{50} 45.0 ± 27 µg/mL), trypomastigote forms of *T. cruzi* (IC_{50} > 90 µg/mL) [24]
V-30	**β-Caryophyllene**	*In vitro* activity against blood stream forms of *T. brucei rhodesiense* STIB 900 strain (IC_{50} 28.9 ± 11.8 µg/mL), trypomastigote forms of *T. cruzi* (IC_{50} 50.1 ± 12.5 µg/mL) [21], *trans*-β-caryophyllene inhibited intracellular amastigotes forms of *T. cruzi* (IC_{50} 24.54 µg/mL), inhibited the epimastigotes of *T. cruzi* (IC_{50} 2.89 µg/mL) [18].
V-39	**ʟ-Linalool**	*In vitro* activity against *T. brucei rhodesiense* (IC_{50} 3.6 ± 2.5 µg/mL), *T. cruzi* (IC_{50} > 90 µg/mL) [21][16], against *T. brucei brucei* bloodstream forms (IC_{50} 2.5 µg/mL; 16.3 µM) [20], *T. cruzi* epimastigotes (IC_{50} 30.16 µg/mL) [18].
V-43	***E*-Nerolidol**	*In vitro* activity against *T. brucei brucei* bloodstream forms (IC_{50} 1.7 µg/mL; 7.6 µM) [25].
V-48	**α-Terpineol**	*In vitro* antiprotozoal activity against blood stream forms of *T. brucei rhodesiense* STIB 900 strain (IC_{50} 0.56 ± 0.4 µg/mL), trypomastigote forms of *T. cruzi* (IC_{50} 61.0 ± 2.1 µg/mL) [16], *T. brucei*/427 (blood stream) HMI-9 medium (ED_{50} 7.1 µg/mL) [25].
V-51	**Borneol**	*In vitro* antiprotozoal activity against blood stream forms of *T. brucei rhodesiense* STIB 900 strain (IC_{50} 24.3 ± 11.8 µg/mL), trypomastigote forms of *T. cruzi* (IC_{50}> 90 µg/mL), bloodstream form of *Trypanosoma b. brucei* TC221 (IC_{50} 70 µg/mL) [26].
V-64	**Citral**	*In vitro* antiprotozoal activity against *T. cruzi* epimastigotes (IC_{50} 37 ± 0.7 µg/mL), *T. cruzi* trypomastigote (IC_{50} 21 ± 1 µg/mL), amastigote (IC_{50} 49 ± 2.3 µg/mL) [19].
V-65	**Citronellal**	Active against *T. brucei [18]*.
V-82	**Carvone**	S-(+)-Carvone *In vitro* antiprotozoal activity on epimastigote of *T. cruzi* (IC_{50} 177 ± 7.9 µg/mL), Trypomastigote of *T. cruzi* (IC_{50} 124 ± 8 µg/mL), amastigote of *T. cruzi* (IC_{50} > 100 µg/mL) [19]
V-92	**Thymol**	*In vitro* activity against blood stream forms of *T. brucei rhodesiense* STIB 900 strain (IC_{50} 0.11 ± 0.01 µg/mL), trypomastigote forms of *T. cruzi* (IC_{50} > 90 µg/mL, bloodstream forms of *T. brucei brucei* STIB 795 in mouse model (*in vivo*) [21]

No.	Compound	Activity [Ref.]
V-105	**Caryophyllene oxide**	(-)-Caryophyllene oxide *in vitro* activity against epimastigotes of *T. cruzi* (IC_{50} 30 ± 1.7 µg/mL), Trypomastigote of *T. cruzi* (IC_{50} 22 ± 0.3 µg/mL), amastigote of *T. cruzi* (IC_{50} 47 ± 1.0 µg/mL) [19]
V-106	**Cineole**	*In vitro* anthelmintic effect against *T. cruzi* epimastigotes (IC_{50} 0.63 µg/mL) [18]
		**3. Compounds with anti-Schistosomal activity**
V-4	**Limonene**	Mixture of (*E*)-anethole and (+)-(*R*)-limonene in the same proportions as in the essential oil affected adult *S. mansoni* worm mortality; all the parasites died upon incubation at a concentration of 100 mg/mL after 24 h and 50 mg/mL after 120 h [27], limonene (43.75 µg/mL) resulted in decreased *S. mansoni* motility continuing until 96 h of observation [28]
V-43	***E*-Nerolidol**	*In vivo* antischistosomal activity in a mouse model of schistosomiasis infected with either adult or juvenile stages of *S. mansoni* [29].
V-90	***trans*-Anethole**	mixture of (*E*)- anethole and (-)-(*S*)-limonene in the same proportions as in the essential oil affected adult of *S. mansoni* worm mortality (50 mg/mL after 120 h and 100 mg/mL after 24 h) [27]
V-107	***E*-Limonene-1,2-oxide**	*In vitro* activity against *S. mansoni* adult worms (100% mortality at 25 µg/mL, 120 h treatment) [30, 31]
		**4. Compounds with anti-Malarial activity (activity against *Plasmodium falciparum*)**
V-4	**Limonene**	*In vitro* activity against *P. falciparum* intraerythrocytic stages (IC_{50} 1.2 mM) [32], human erythrocytes exposed to chloroquine-resistant *P. falciparum* K1 strain (IC_{50} 18.6 ± 2.19 µg/mL) [33]
V-5	**γ-Terpinene**	*In vitro* antiprotozoal activity against erythrocytic stages of *P. falciparum* (IC_{50} > 20 µg/mL) [21]
V-6	***α*-Terpinene**	*In vitro* activity against *P. falciparum* (IC_{50} 3.7 ± 1.5 µg/mL) [21]
V-11	**α-Pinene**	*In vitro* antiprotozoal activity against erythrocytic stages of *P. falciparum* (IC_{50} 10.7 ± 1.2 µg/mL) [21], *P. falciparum*/FCR-3 (IC_{50} 1.2 µM; additive and synergistic interaction with linalyl acetate and carvacrol, respectively on the in vitro growth of *P. falciparum*) [34]
V-19	***P*-Cymene**	*In vitro* antiprotozoal activity against erythrocytic stages of *P. falciparum* (IC_{50} > 20 µg/mL) [21]
V-30	**β-Caryophyllene**	In vitro antiprotozoal activity against erythrocytic stages of *P. falciparum* (IC_{50} 12.8 µg/mL) [21]
V-39	**L-Linalool**	*In vitro* antiprotozoal activity against and *P. falciparum* (IC_{50}> 90 µg/mL) [21], against *P. falciparum* intraerythrocytic stages (IC_{50} 0.28 mM) [32]
V-43	***E*-Nerolidol**	*In vitro* activity against *P. falciparum* intraerythrocytic stages (IC_{50} 1.2 mM) [32] and *in vivo* activity against mouse model infected with *P. berghei* ANKA [30]
V-48	**α-Terpineol**	*In vitro* antiprotozoal activity against erythrocytic stages of *P. falciparum* (IC_{50} > 20 µg/mL) [21]
V-51	**Borneol**	*In vitro* activity against erythrocytic stages of *P. falciparum* (IC_{50}> 90 µg/mL) [21].
V-91	**Estragole**	*in vitro* activity of against the human malaria parasite *P. falciparum* (K1 strain) (IC_{50} 30.70 µg/mL) [35]
V-92	**Thymol**	*in vitro* activity against *P. falciparum* (K1 strain) (IC_{50} 4.50 µg/mL) [31], erythrocytic stages of *P. falciparum* (IC_{50} 5.7 ± 0.03 µg/mL) [21]
V-118	***n*-Hexadecanoic acid**	Antiplasmodial activity effect (IC_{50} 23.3 ± 3.14 µg/mL) [33]
		**5. Compounds with anti-leishmania activity (activity against *Leishmania sp*.)**
V-4	**Limonene**	*In vitro* activity of R(+)-Limonene against promastigotes of *L. mexicana mexicana* (IC_{50} 16.59 µg/ml) [36], promastigotes of *L.* (Viannia) *braziliensis* (IC_{50} 876.2 ± 216 µM) [20], *L. major* (IC_{50} 16.0 ± 0.21 µg/mL) [37], *L. amazonensis* (IC_{50} 278 ± 12 µM µM) [38], promastigotes of *L. amazonensis* (IC_{50} 549 ± 169 µM) [39].

No.	Compound	Activity [Ref.]
		Limonene killed *L. amazonensis* promastigotes (IC_{50} 252.0 ± 49.0 µM) and amastigotes (IC_{50} 147.0 ± 46.0 µM), against the intracellular amastigote of *L. major* (IC_{50} 21.3 ± 2.15 µg/mL) [40].<br>The Limonene-carvacrol combination n (5:0; 1:1; 1:4; 2:3; 3:2; 4:1, and 0:5) showed potential antileishmania activity against promastigotes forms of *L. major* (IC_{50} 5.8 to 19.0 µg/mL) [37].<br>The treatment with the terpenes limonene reduced the infection rates of *L. amazonensis*-infected macrophages by 78%. *L. amazonensis*-infected mice treated with limonene (intrarectally or topically) displayed a significant reduction in lesion size and parasite load [40].
V-5	**γ-Terpinene**	*In vitro* antiprotozoal activity against amastigotes of *L. donovani* (strain MHOM/ET/67/L82) (IC_{50} > 90 µg/mL) [21]
V-6	**α-Terpinene**	*In vitro* activity against amastigotes of *L. donovani* (strain MHOM/ET/67/L82 (IC_{50} 10.5 ± 1.7 µg/mL) [21]
V-8	**Terpinolene**	Antileishmanial activity against promastigote forms *L. tropica* (MHOM/AF/88/KK27) (IC_{50} 11.6 µg/mL) [41]
V-11	**α-Pinene**	*In vitro* activity against promastigotes of *L. infantum* (IC_{50} 45.94 µg/mL) [13], amastigotes *L. donovani* (strain MHOM/ET/67/L82) (IC_{50} 81.9 ± 9 µg/mL) [21]
V-13	**Camphene**	Quantitative structure–activity relationship (QSAR) study of Camphene indicated its antiparasitic activity against *L. donovani* (IC_{50} = 4.2 µM) [23]
V-19	***P*-Cymene**	*In vitro* antiprotozoal activity against amastigotes of *L. donovani* (strain MHOM/ET/67/L82) (IC_{50} > 90 µg/mL) [21]
V-23	**α-Humulene**	*In vitro* activity against *Leishmania donovani* promastigotes (IC_{50} 19 µg/µL) [42]
V-29	**δ-Cadinene**	*In vitro* activity against promastigotes of *L. donovani* (IC_{50} 4 µg/mL; IC_{90} 7 µg/mL) [42]
V-30	**β-Caryophyllene**	*In vitro* antiprotozoal activity against amastigotes of *L. donovani* (strain MHOM/ET/67/L82) (IC_{50} 52.4 ± 17.4 µg/mL) [16], promastigotes stages of *L. amazonensis* (IC_{50} 96 ± 19 µM) [38]<br>*trans*-β-caryophyllene inhibited *L. infantum* (IC_{50} 53.39 µg/mL), inhibited the epimastigotes of and *L.* the *infantum* (IC_{50} 24.02 µg/mL) [18]
V-39	**Linalool**	Linalool on Glutathione modified Gold Nanoparticles displayed highly effective on the parasite amastigote phase of *L. tropica* at 10 µg/mL [43]<br>*In vitro* antiprotozoal activity against promastigotes of *L. amazonensis* (LD_{50} value of 4.3 ng/mL) [44]
V-43	***E*-Nerolidol**	*In vitro* anthelmintic effect against, *L. amazonensis* promastigotes (IC_{50} 7.9 µM) [39]<br>*In vitro* anthelmintic effect of nerolidol (mixture of *cis*- and *trans*-nerolidol) against promastigotes of *L. amazonensis* (IC_{50} 85 µM), *L. braziliensis* (IC_{50} 74 µM), and *L. chagasi* (IC_{50} 75 µM) and amastigotes of *L. amazonensis* (IC_{50} 67 µM) [45].<br>(mixture of *cis*- and *trans*-nerolidol) active against intracellular amastigotes; the treatment of *L. amazonensis*-infected macrophages cultures 100 µM nerolidol resulted in 95% reduction in infection rates [45]
V-48	**α-Terpineol**	*In vitro* antiprotozoal activity against *L. amazonensis* promastigotes (IC_{50} 678 ±59 µM) [35], amastigotes of *L. donovani* (strain MHOM/ET/67/L82) (IC_{50} 75.9 ± 4.7 µg/mL) [21]
V-51	**Borneol**	*In vitro* antiprotozoal activity against amastigotes of *L. donovani* (strain MHOM/ET/67/L82) (IC_{50} 52.1 ± 16.6 µg/mL) [45].
V-55	**α-Bisabolol**	*in vitro* activity against amastigotes of *L. amazonensis* 16.0 µg/mL and L. infantum 9.5 µg/mL (IC_{50} 0.01 µg/mL; 44.9 nM) [44]
V-58	**Guaiol**	*in vitro* activity against amastigotes of *L. amazonensis* (IC_{50} 0.01 µg/mL; 44.9 nM) [44, 46]
V-64	**Citral**	*in vitro* activity against amastigotes of *L. amazonensis* (IC_{50} 25 µg/mL) [44]

No.	Compound	Activity [Ref.]
V-91	**Estragole**	*In vitro* inhibition activity against promastigotes of *L. tarentolae* (IC_{50} 28.5 µg/mL) and *L. amazonensis* (IC_{50} 25.5 µg/mL, respectively) and amastigotes of *L. amazonensis* (IC_{50} 1.4 ± 0.1 µg/mL) [47, 48]
V-92	**Thymol**	*In vitro* activity against amastigotes of *L. donovani* (strain MHOM/ET/67/L82) (IC_{50} 17.3 ± 4.1 µg/mL) [21]
V-105	**Caryophyllene oxide**	Both β-caryophyllene oxide and the 1:4 mixture of lupenone and βcaryophyllene oxide showed activity against promastigotes of *L. amazonensis*, *L. mexicana*, *L. braziliensis*, *L. aethiopica* and *L. tropica* with IC_{50} values ranging from 14.0 to 39.3 µg/mL [44]
V-106	**Cineole**	*In vitro* anthelmintic effect against, *L. amazonensis* promastigotes (IC_{50} 4697 µM) [39]
V-118	***n*-Hexadecanoic acid**	Active against the intracellular amastigote of *L. major* (MRHO/IR/75/ER), (IC_{50} 19.6 ± 3.05 µg/mL) [33]
		**6. Compounds with anti-taxoplasmosis activity**
V-91	**Estragole**	*In vitro* activity against *Toxoplasma gondii* ME49 strain in a congenital and noncongenital model of toxoplasmosis [49]
V-92	**Thymol**	*In vitro* activity against *T. gondii* ME49 strain in a congenital and noncongenital model of toxoplasmosis [49]
		**7. Compounds with anti-fish parasitic activity**
V-5	**γ-Terpinene**	*In vitro* efficacy against ancyrocephalid monogeneans found on farmed Nile tilapia (*Oreochromis niloticus*); 86% mortality rate at concentration of 36 mg/L and 5 h treatment [50]
V-11	**α-Pinene**	*In vitro* antiprotozoal activity against *Dactylogyrus minutus* (M***onogenea***); 100% mortality in the concentration of 90 g/L at 1 h treatment [51]<br>*In vitro* antiprotozoal activity against different stages of *Ichthyophthirius multifiliis*; 100% mortality of both the trophont and tomont stages at concentration of 84.7 mg/ L [51]
V-12	**β-Pinene**	(+)*β*-Pinene showed 100% mortality against *Dactylogyrus minutus* in the concentration of 90 g/L at 1 h. while (-)*β*-Pinene showed 100% mortality in the concentration of 8g/L at 1 h. [48]
V-14	**Sabinene**	*In vitro* antiprotozoal activity against different stages of *Ichthyophthirius multifiliis*; 100% mortality of both the trophont and tomont stages at concentration of 77.34 mg/ L [48]
V-30	**β-Caryophyllene**	*In vitro* antiprotozoal activity of the mixture of (*E*)-Caryophyllene + epiglobulol against different stages of *Ichthyophthirius multifiliis* [52]
V-41	**Geraniol**	*In vitro* larvicidal activity against the fish parasite *Contracaecum sp* (parasite of the fish *Hoplias malabaricus*) (LD_{50} 182.38; LD_{100} 470.93 in 48 h) [53, 54]
V-94	**Eugenol**	*In vitro* antiparasitic activity against the fish parasite *Gyrodactylus sp*. (parasite of the fish *Rhamdia quelen*) at concentration of 5 mg/mL and I h exposure and efficacy (mortality of the parasite) of 80% and efficacy of 90% at concentration of 10 mg/ mL [24, 53]
V-102	***cis*-Limonene oxide**	*In vitro* efficacy against *Ancyrocephalid monogeneans* found on farmed Nile tilapia (*Oreochromis niloticus*); 90% mortality rate at concentration of 55.4 mg/L and 5 h treatment [50]
V-106	**Cineole**	*In vitro* antiparasitic activity against *Dactylogyrus minutus* (100% mortality in the concentration of 1g/L at 1 h treatment) [52]
		**8. Compounds with activity affecting parasite of stored grains**
V-1	**β-Myrcene**	Fumigant toxicity against *S. granarius* adults: (96 97% mortality after 96 h exposure in the dosage of 7.9) [55]
V-4	**D-Limonene**	Fumigant toxicity against *Tribolium castaneum* adults (LC_{50} 6.2 mg/L air), Contact toxicity against *Tribolium castaneum* (LC_{50} 15 µg/adults) [56].<br>*In vitro* toxicity against adults of *Sitophilus zeamais* adults LD_{50} 15.0 µL (after 96 h) [57]

No.	Compound	Activity [Ref.]
		Fumigant toxicity against *S. granarius* adults: 92.9, 97and 100% mortality in the dosage of 16.8 µg after 24, 48 and 72 h exposure, respectively and 91.9 and 96% mortality in the dosage of 8.4 µg after 72 and 96 h exposure, respectively [55].
V-5	**γ-Terpinene**	Fumigant toxicity against *Tribolium castaneum* adults (LC_{50} 0.195 mg/cm^3, 24 h after treatment [58]<br>Fumigant toxicity against *S. granarius* adults (100% mortality in the dosage of 17.0 µg after 24 48 h. exposure and 87.9% mortality in the dosage of 8.5 µg after 96 h. exposure [55].
V-11	**α-Pinene**	*In vitro* fumigant toxicity against *Tribolium castaneum* adults (LC_{50} 115 µL/L) [58], against *Sitophilus zeamais* adults (LD_{50} 32.4 µL after 96 h) [57], against *S. granarius* adults (100% mortality in the dosage of 9.5 µg after 24h. exposure [55].
V-12	**β-Pinene**	*In vitro* insecticidal toxicity against *Sitophilus zeamais* adults (LD_{50} 26.4 µL, after 96 h) [57]<br>Fumigant toxicity against *Tribolium castaneum* (LC_{50} 88 µL/L) [58]<br>Fumigant toxicity against *S. granarius* adults: 92.9 and 100% mortality in the dosage of 8.7 µg after 72 and 96 h. exposure, respectively [55]
V-15	**δ-3-Carene**	*In vitro* insecticidal toxicity against *Sitophilus zeamais* adults LD_{50} 4.8 µL(after 96 h) [57]<br>Toxic against first instars larvae of Colorado potato beetle (% of mortality 100% after 96 h, 10 µl) [59]<br>Fumigant toxicity against *S. granarius* adults: caused 91.9 and 100% mortality in the dosage of 17.4 µg after 72, 96 h. exposure, respectively and 88.9 and 100% mortality in the dosage of 8.7 µg after 72, 96 h. exposure, respectively [55]
V-13	**Camphene**	Fumigant activity against *Sitophilus zeamais* adults (LC_{50} 95 µL/L, 48 h exposure) [60], (LC_{50} 28.8 µL, 96 h exposure) [57]<br><br>Fumigant activity against *Tribolium castaneum* adults (LC_{50} 118 µL/L; 6.2 mg/L air) [56, 60]; contact toxicity against adults of *Tribolium castaneum* (IC_{50} 21.6 µg/adult) [56]<br><br>Fumigant toxicity against rice *Weevil Sitophilus oryzae* (100% mortality, 12.5 mg/L air concentration) [61], (100% mortality, concentration of 8.7 µg, 96 h exposure) [55]<br><br>Contact toxicity against adults of *Lasioderma serricorne* (LS) adults (IC_{50} 19.9 µg/adult) [56].
V-19	***P*-Cymene**	Fumigant toxicity against *Tribolium castaneum* adults (LC_{50} 0.140 mg/cm^3, 24 h treatment) [58]
V-23	**α-Humulene**	Toxic to *Sitophilus zeamais* adults (IC_{50} 20 µg sample/mg of insect, 7 d exposure at 30°C), *Tribolium castaneum* (IC_{50} 31 µg sample/mg of insect) [62]
V-39	**Linalool**	Fumigant activity against *Sitophilus zeamais* adults (LD_{50} 2.4 µL, 96 h exposure) [57]<br>Fumigant toxicity against *S. granarius* adults: (100% mortality, 8.6 µg, 24 h. exposure) [55]
V-40	**Citronellol**	Fumigant activity against *Sitophilus zeamais* adults LD_{50} 21.9 µL(after 96 h) [57]
V-41	**Geraniol**	Contact toxicity against *Sitophilus oryzae* (LC_{50} 28.76 µg/cm^2) [63]
V-48	***α*-Terpineol**	Fumigant activity against *Sitophilus zeamais* adults (LD_{50} 7.7 µL, 96 h exposure) [57]; *S. granarius* adults (97% mortality, dosage of 20 µg, 24 h. exposure) [55]; *Liposcelis bostrychophila* adult (LC_{50} 0.58 mg/L air), *Lasioderma serricorne* adult (LC_{50} 3.27 mg/L air) [64]<br>Contact toxicity against *Liposcelis bostrychophila* adult (LC_{50} 37.76 µg/cm), *Lasioderma serricorne* adult (LC_{50} 11.99 µg /adult) [64]
V-51	**Borneol**	Fumigant activity against *Sitophilus zeamais* adults (LD_{50} 8.9 µL, 96 h exposure) [57]
V-65	**Citronellal**	Fumigant activity against *Sitophilus zeamais* adults (LD_{50} 8.1 µL, 96 h exposure) [57]

No.	Compound	Activity [Ref.]
		Toxic against first instars larvae of Colorado potato beetle (100% of mortality, after 96h exposure, 20 µl) [59]
V-72	***p*-Anisaldehyde**	Fumigant toxicity against *S. granarius* adults: (100% mortality, of 11.2 µg, 24h. exposure) [55].
V-73	**Cuminaldehyde**	Fumigant toxicity against *S. granarius* adults (93.9 and 100% mortality in the dosage of 9.8 µg after 24 and 48 h. exposure, respectively and 100% mortality in the dosage of 19.6 µg after 24 h. exposure [55], Toxic against *Sitophilus oryzae* adults (fumigant toxicity, LC_{50} 71.4 mg/L) and Contact toxicity(LC_{50} =32.59 µg/cm^2) [63] . Contact toxicity against *Tribolium castaneum* with (LC_{50} 42.08 µg/cm^2) [63]
V-80	***cis*-Dihydrocarvone**	Fumigant toxicity against *S. granarius* adults (100% mortality, 9.3 µg, 24 h. exposure) [55]
V-82	**Carvone**	- Fumigant toxicity against *S. granarius* adults (100% mortality, 9.6 µg, 24h. exposure) [55]<br>(-)-carvone fumigant toxicity against *T. pisana* (LC_{50} 27.01 mg/L), *S. littoralis* (LC_{50} 19.48 mg/L) [65]<br>- Toxic against *Sitophilus oryzae* adults (fumigant toxicity, LC_{50} 17.87 mg/L) and Contact toxicity (LC_{50} 28.17 µg/cm^2) [63]<br>- Toxic against *Tribolium castaneum* adults (fumigant toxicity, LC_{50} 17.87 mg/L) and Contact toxicity (LC_{50} 75.22 µg/cm^2) [63]
V-85	**Fenchone**	Fumigant activity against *Sitophilus zeamais* adults (LD_{50} 2.4 µL, 96 h exposure) [57]<br>Fumigant toxicity against *S. granarius* adults (100% mortality, 9.5 µg, 24h. exposure) [55]
V-86	**Camphor**	- Fumigant toxicity against *Sitophilus zeamais* adults (LD_{50} 4.9 µL, after 96 h) [53] (LC_{50} 78 µL/L, 48 h exposure) [60].<br>- Fumigant toxicity against *S. granarius* adults (100% mortality, dosage of 20 µg, 72 h. exposure) [51][55]<br>-Fumigant toxicity against *Tribolium castaneum* adults (LC_{50} $<$ 2.3 mg/L air) [52], (LC_{50} 36 µL/L, 48 h exposure) [60]
V-90	***trans*-Anethole**	Fumigant toxicity against *S. granarius* adults (100% mortality, 10 µg, after 96 h. exposure and 100% mortality in the dosage of 20 µg after 48 h. exposure [55]
V-92	**Thymol**	Fumigant toxicity against *Tribolium castaneum* adults (LC_{50} 1.2 µg/cm^3, 24 h treatment) [58].<br>Fumigant toxicity against *S. granarius* adults (86.9% mortality, dose of 20 µg after 72 h. exposure) [55]
V-94	**Eugenol**	Fumigant toxicity against *S. granarius* adults (100% mortality, 10.6 µg, 72 h. exposure) [55]
V-102	**Limonene oxide**	- Fumigant toxicity against *Sitophilus zeamais* adults (LD_{50} 3.2 µL, 96 h exposure) [57]<br>-Fumigant toxicity against *S. granarius* adults (100% mortality, dosage of 9.3 µg after 24 h. exposure) [55]
V-105	**Caryophyllene oxide**	Fumigant toxicity against *Tribolium castaneum* adults (LC_{50} 0.18 µg/cm^3, 24 h treatment) [58]
V-106	**Cineole**	-Toxic to *Sitophilus zeamais* adults: (contact toxicity, LC_{50} = 48 µL/L, 7 days, 30 °C) [58][62]; fumigant activity (LD_{50} 1.9 µL, 96 h exposure) [57]<br>- Toxic to *Tribolium castaneum* adults (contact toxicity, IC_{50} 24 µg sample/mg of insect, after 7 d at 30 °C) [58]; fumigant toxicity against adults *T. castaneum* (LC_{50} 17.16 mg/l) [59]; fumigant toxicity (LC_{50} 41 µL/L, 48 h exposure, 30 °C) [60]<br>-Contact toxicity against adults *S. oryzae* (LC_{50} 14.19 mg/l) [63]<br>-Fumigant toxicity against *S. granarius* adults (100% mortality, dosage of 9.2 µg, 48 h. exposure) [55]
V-109	**Geranyl acetate**	Fumigant activity against *Sitophilus zeamais* adults (LD_{50} 11.8 µL, 96 h exposure) [57]
		**9. Compounds with activity affecting parasite of animals (cattle and sheep)**
V-4	**Limonene**	*In vitro* anthelmintic activity (egg hatch assay) against *Haemonchus contortus* (LC_{50} 207.5 mg/ml) [66]

No.	Compound	Activity [Ref.]
V-39	**Linalool**	*In vitro* anthelmintic activity (egg hatch assay) against *Haemonchus contortus* (LC_{50} 0.29 mg/mL) [66]<br>*In vitro* acaricidal activity against *Hyalomma scupense*: adults (LC_{50} 0.97 mg/ml), larvae (LC_{50} 1.19 mg/ml), acetylcholinestrase inhibitor with IC_{50} of 0.42 mg/mL for *H. scupense* and IC_{50} of 0.201 mg/mL for *He. polygyrus* larvae [67]<br>*In vitro* anthelmintic activity (larval motility assay, on the third-stage larvae) against *Trichostrongylus colubriformis* nematode, *Trichostrongylus axei*, *Teladorsagia circumcincta*, *Trichostrongylus vitrines*, and *Haemonchus contortus* a with (IC_{50} ranged from 0.51 to 1.76%) [68]
V-43	***E*-Nerolidol**	*in vitro* activity against the growth of *Babesia bovis*, (IC_{50} 21.0 µM), *B. bigemina* (29.6 µM), *B. ovata* (26.9 µM), and *B. caballi* 23.1 µM [69].
V-82	**Carvone**	*In vitro* anthelmintic activity (egg hatch assay) against *Haemonchus contortus* (LC_{50} 0.085 mg/mL) [66], *in vivo* significantly reduced fecal egg count, decreased male length, and reproductive capacity of female [70, 71].
V-86	**Camphor**	*In vitro* anthelmintic activity against larvae of *Haemonchus contortus* (LC_{50} 7.80 mg/ mL) [72]
V-90	***trans*-anethole**	*In vitro* anthelmintic activity against *Haemonchus contortus*: Ovicidal EC_{50} (0.69 mg/mL); Larvicidal (EC_{50} 2.11) mg/mL [66, 70, 71, 73].<br>*In vivo* reduced fecal egg count, decreased male length, and reproductive capacity of female at 50 mg/kg concentration [70, 71].
V-91	**Estragole**	*in vitro* acaricidal activity against *Hyalomma scupense*: adults (LC_{50} 0.73 mg/mL) and larvae (LC_{50} 0.22 mg/mL) [67]
V-92	**Thymol**	*In vitro* activity against *Haemonchus contortus*: Ovicidal (EC_{50} 0.13-0.55 mg/mL) [66, 73]; Larvicidal (EC_{50} 2.49 mg/ml [73].<br>*in vitro* acaricidal activity against *Rhipicephalus microplus* tick larvae (LC_{50} 0.5–1.0%) [74]
V-94	**Eugenol**	*in vitro* activity against *Haemonchus contortus* based on (LC_{50} 0.57 mg/mL) (in vitro egg hatch assay) [66].<br>*in vitro* acaricidal activity of eugenol against *Rhipicephalus microplus* tick larvae (LC_{50} 0.25–0.5%) [74]
V-97	**Elemicin**	*in vitro* acaricidal activity of elemicin against *Rhipicephalus microplus* tick larvae (LC_{50} 0.125–0.25%) [74]
V-106	**Cineole**	*In vitro* anthelmintic activity against *Haemonchus contortus* (LC_{50} 4.74 mg/mL) (*in vitro* egg hatch assay) [66], Inhibit the larval development (LC_{50} 5.07 mg/ mL), inhibited 60.3% of larval migration at 10 mg/mL [72]
	**10. Compounds with anti-*pediculus humanus* activity**	
V-4	**Limonene**	Toxic to KR-HL Females of *P. h. capitis* (LT_{50} 52.9 min, using Contact + Fumigant Mortality Bioassay during a 3h Exposure to 0.25 mg/cm^2) [75].
V-5	**γ-Terpinene**	Toxic to KR-HL Females of *P. h. capitis* (LT_{50} 60.3 min, using Contact + Fumigant Mortality Bioassay during a3h Exposure to 0.25 mg/cm^2) [75].<br>Toxic to Females of *P. h. capitis* using Contact + Fumigant Mortality bioassay, 12 exposure): KR-HL strain (IC_{50} to 0.129 mg/cm^2), BR-HL Strains (IC_{50} to 0.122 mg/cm^2); ovicidal activity against BR-HL Eggs (28% egg hatch inhibition on 24 h exposure) [75].
V-11	*α*-Pinene	Toxic to KR-HL Females of *P. h. capitis* (LT_{50} 26.5 min, using Contact + Fumigant Mortality Bioassay during a3h Exposure to 0.25 mg/cm^2) [75].
V-11	*β*-Pinene	Toxic to KR-HL Females of *P. h. capitis* (LT_{50} 29.4 min, using Contact + Fumigant Mortality Bioassay during a3h Exposure to 0.25 mg/cm^2) [75].<br>;.
V-13	**Camphene**	Toxic to KR-HL Females of *P. h. capitis* (LT_{50} 37.8 min, using Contact + Fumigant Mortality Bioassay during a3h Exposure to 0.25 mg/cm^2) [75].
V-19	***P*-Cymene**	Toxic to KR-HL Females of *P. h. capitis* (LT_{50} 50.9 min, using contact + fumigant mortality bioassay, 3 h exposure to 0.25 mg/cm^2) [75].
V-39	**Linalool**	Fumigant mortality against adult female *Pediculus humanus capitis* (LT_{50} 15.4 min at a concentration of 0.25 mg/cm^2), using contact + fumigant mortality bioassay, 3 h exposure to 0.25 mg/cm^2); toxic to

No.	Compound	Activity [Ref.]
		females of *P. h. capitis* (using contact + fumigant mortality bioassay, 12 exposure): KR-HL strain (IC_{50} to 0.035 mg/cm^2), BR-HL Strains (IC_{50} to 0.040 mg/cm^2); ovicidal activity against BR-HL Eggs (100% egg hatch inhibition on 24 h exposure, 0.5 mg/cm^2) [75]
V-48	**α-Terpineol**	Fumigant mortality against adult female *Pediculus humanus capitis* (LT_{50} 26.6 min at a concentration of 0.25 mg/cm^2), using contact + fumigant mortality bioassay, 3 h exposure to 0.25 mg/cm^2); toxic to females of *P. h. capitis* (using contact + fumigant mortality bioassay, 12 exposure): KR-HL strain (IC_{50} to 0.045 mg/cm^2), BR-HL Strains (IC_{50} to 0.043 mg/cm^2); ovicidal activity against BR-HL Eggs (89% egg hatch inhibition on 24 h exposure, 0.5 mg/cm^2) [75]
V-86	**Camphor**	Fumigant mortality against adult female *Pediculus humanus capitis* (LT_{50} 34.2 min at a concentration of 0.25 mg/cm^2), using contact + fumigant mortality bioassay, 3 h exposure to 0.25 mg/cm^2); Toxic to Females of *P. h. capitis* (using contact + fumigant mortality bioassay, 12 exposure): KR-HL strain (IC_{50} to 0.022 mg/cm^2), BR-HL Strains (IC_{50} to 0.020 mg/cm^2) [75]
V-106	**Cineole**	Fumigant mortality against adult female *Pediculus humanus capitis* (LT_{50} 14.1 min at a concentration of 0.25 mg/cm^2), using contact + fumigant mortality bioassay, 3 h exposure to 0.25 mg/cm^2); Toxic to Females of *P. h. capitis* using contact + fumigant Mortality bioassay, 12 exposure): KR-HL strain (IC_{50} to 0.068 mg/cm^2), BR-HL Strains (IC_{50} to 0.066 mg/cm^2) [75]
		**11. Compounds with anti-*Musca domestica* (housefly) activity**
V-1	**β-Myrcene**	*In vitro* activity against adults of *Musca domestica* (LC_{50} 4.95 µL/L in 24 h treatment) [76]
V-4	**Limonene**	*In vitro* activity against adults of *Musca domestica* (LC_{50} 3.2 µL/L in 24 h treatment) [76]
V-5	**γ-Terpinene**	*In vitro* activity against adults of *Musca domestica* (LC_{50} 2.1 µL/L in 24 h treatment) [76]
V-6	**α-Terpinene**	*In vitro* activity against adults of *Musca domestica* (LC_{50} 2.4 µL/L in 24 h treatment) [76]
V-8	**Terpinolene**	*In vitro* activity against adults of *Musca domestica* (LC_{50} 1.8 µL/L in 24 h treatment) [76]
V-11	**α-Pinene**	*In vitro* activity against adults of *Musca domestica* (LC_{50} 2.6 µL/L in 24 h treatment) [76]
V-12	**β-Pinene**	*In vitro* activity against adults of *Musca domestica* (LC_{50} 2.0 µL/L in 24 h treatment) [76]
V-13	**Camphene**	*In vitro* activity against adults of *Musca domestica* (LC_{50} 6.1 µL/L in 24 h treatment) [76]
V-15	**δ-3-Carene**	*In vitro* activity against adults of *Musca domestica* (LC_{50} 5.0 µL/L in 24 h treatment) [76]
V-19	***P*-Cymene**	*In vitro* activity against adults of *Musca domestica* (LC_{50} 0.7 µL/L in 24 h treatment) [76]
V-39	**Linalool**	*In vitro* activity against adults of *Musca domestica* (LC_{50} 4.4 µL/L in 24 h treatment) [76]
V-40	**Citronellol**	*In vitro* activity against adults of *Musca domestica* (LC_{50} 5.5 µL/L in 24 h treatment) [76]
V-41	**Geraniol**	*In vitro* activity against adults of *Musca domestica* (LC_{50} 4.6 µL/L in 24 h treatment) [76]
V-47	**α- Terpineol**	*In vitro* activity against adults of *Musca domestica* (LC_{50} 3.7 µL/L in 24 h treatment) [76]
V-51	**Borneol**	*In vitro* activity against adults of *Musca domestica* (LC_{50} 7.7 µL/L in 24 h treatment) [76]
V-65	**Citronellal**	*In vitro* activity against adults of *Musca domestica* (LC_{50} 1.8 µL/L in 24 h treatment) [76]

No.	Compound	Activity [Ref.]
V-73	**Cuminaldehyde**	*In vitro* activity against adults of *Musca domestica* (LC_{50} 0.6 µL/L in 24 h treatment) [76]
V-82	**Carvone**	*In vitro* activity against adults of *Musca domestica* (LC_{50} 1.7 µL/L in 24 h treatment) [76]
V-85	**Fenchone**	*In vitro* activity against adults of *Musca domestica* (LC_{50} 2.2 µL/L in 24 h treatment) [76]
V-86	**Camphor**	*In vitro* activity against adults of *Musca domestica* (LC_{50} 1.9 µL/L in 24 h treatment) [76]
V-92	**Thymol**	*In vitro* activity against adults of *Musca domestica* (LC_{50} 1.6 µl/L in 24 h treatment) [76]
V-94	**Eugenol**	*In vitro* activity against adults of *Musca domestica* (LC_{50} 6.9 µL/L in 24 h treatment) [76]
V-106	**Cineole**	*In vitro* activity against adults of *Musca domestica* (LC_{50} 3.3 µL/L in 24 h treatment) [76]
V-109	**Geranyl acetate**	*In vitro* activity against adults of *Musca domestica* (LC_{50} 4.2 µL/L in 24 h treatment) [76]
V-110	**Neryl acetate**	*In vitro* activity against adults of *Musca domestica* (LC_{50} 4.1 µL/L in 24 h treatment) [76]
		**12. Compounds with anti-house mosquitoes (*Culex pipiens*)**
V-1	**β-Myrcene**	*In vitro* adulticidal activity against adults of *Culex pipiens* L. (93.3% mortality at 100 mg/filter after 48 h treatment) [77]
V-4	***S*-(-)-limonene**	*In vitro* adulticidal activity against adults of *Culex pipiens* L. (96.7% mortality at 100 mg/filter after 48 h treatment) [77]
V-41	**Geraniol**	*In vitro* larvicidal activity against *Culex pipiens* L. (IC_{50} 38.6 mg/L after 24 h treatment) [77]
V-73	**Cuminaldehyde**	*In vitro* larvicidal activity against *Culex pipiens* L. (IC_{50} 38.9 mg/L after 24 h treatment) [77]
V-82	**(*S*)-(+)-carvone**	*In vitro* adulticidal activity against adults of *Culex pipiens* L. (100% mortality at 100 mg/filter after 48 h treatment) [77]
V-85	**(*S*)-Fenchone**	*In vitro* adulticidal activity against adults of *Culex pipiens* L. (96.7% mortality at 100 mg/filter after 48 h treatment) [77]
V-86	**(*R*)-camphor**	*In vitro* adulticidal activity against adults of *Culex pipiens* L. (96.7% mortality at 100 mg/filter after 48 h treatment) [77]
V-92	**Thymol**	*In vitro* adulticidal activity against adults of *Culex pipiens* L. (96.7% mortality at 100 mg/filter after 48 h treatment) [77]
		**Compounds with Antiparasitic activity against plant parasites**
V-1	**β-Myrcene**	Fumigant toxicity against Two-spotted spider mite, *Tetranychus urticae* (IC_{50} 5.77 mg/L) [78], *T. pisana* (LC_{50} 3.8 mg/L) and *S. littoralis* (LC_{50} 2.2 mg/L) [65]<br>Toxic against first instars larvae of Colorado potato beetle (% of mortality 100% after 72 h, 20 µl) [59]
V-4	(-)-**Limonene**	Contact toxicity against *Lasioderma serricorne* (LC_{50} 13.7 µg/adults) adults [56].<br>Toxic against first instars larvae of Colorado potato beetle (% of mortality 90% after 96 h, 10 µl) [59]<br>(-)limonene Fumigant toxicity against Two-spotted spider mite, *Tetranychus urticae* (IC_{50}) 8.09 mg/L [78], *T. pisana* (LC_{50} 8.39 mg/L) and *S. littoralis* with (LC_{50} 11.36 mg/L) [65]
V-6	**γ-Terpinene**	Toxic against first instars larvae of Colorado potato beetle (% of mortality 100% after 12 h, 20 µl) [59]
V-11	**α-Pinene**	Fumigant activity on second instar larvae of *Spodoptera frugiperda* (LC_{50} 5.7 µg/L of air) [79]

No.	Compound	Activity [Ref.]
V-12	**β-Pinene**	Fumigant activity on second instar larvae of *Spodoptera frugiperda* (LC_{50} 14.0 µg/L of air) [79]<br>Toxic against first instars larvae of Colorado potato beetle (% of mortality 100% after 24 h, 20 µl) [59]
V-13	**Camphene**	Insecticidal activity on different stages of *Helicoverpa armigera*: contact toxicity (LD50 5.13 µg/adult), Larvicidal activity Mortality 100.0 ± 0.0% at a concentration of 25 µg/mL, LC_{50} = 10.64 µg/mL), Ovicidal activity Egg hatchability 55.1 ± 0.8% at a concentration of 150 µg/mL, EC50 = 35.39 µg/mL) [80]<br>Fumigant toxicity on *Liposcelis bostrychophila* (LC_{50} 10.1 mg/L air) [81]<br>Fumigant toxicity on different stages of *Rhynchophorus ferrugineus*: Egg (LC_{50} 34.1 µL/L), adults (LC_{50} 99.9 µL/L), 4th larval instar (LC_{50} 46.9 µL/L) [23]<br>Fumigant toxicity on two-spotted spider mite, *Tetranychus urticae* Koch (LC_{50} 61.45 mg/l) [78]<br>Fumigant activity on second instar larvae of *Spodoptera frugiperda* (LC_{50} < 0.6 µg/L of air) [79]<br>Insecticidal activity on *Spodoptera litura* 4th instar larvae (LC_{50} = 6.28 µg/mL) [82]<br>Toxic against different stages of Colorado potato beetle (*Leptinotarsa decemlineata* Say) at concentration of 20 mg: adults (Mortality = 76.7 ± 23.3%), first instars larvae of (Mortality = 26.7 ± 6.7%), second instars larvae of (Mortality = 73.3 ± 8.8%), third instar larvae (Mortality = 50.0 ± 5.8%) [59]<br>Fumigant toxicity against adults of *Theba pisana* (LC_{50} 33.53 mg/L) and not active against contact toxicity (LD50 > 1000 µg/snail) [65]
V-23	**α-Humulene**	Larvicidal activity on 4th instar larvae of *Spodoptera litura* (LC_{50} 12.9) µg/mL [82]<br>Active against different stages of *Helicoverpa armigera*: third instar larvae (LC_{50} 20.8 µg/mL), Ovicidal activity (EC_{50} 77.1 µg/mL) [80]
V-39	**Linalool**	Toxic against first instars larvae of Colorado potato beetle (% of mortality 100% after 72 h, 20 µl) [59]<br>(-)-linalool Fumigant toxicity against Two-spotted spider mite, *Tetranychus urticae* (IC_{50} 0.56 mg/L) [78]
V-41	**Geraniol**	Fumigant toxicity against Two-spotted spider mite, *Tetranychus urticae* (IC_{50} 4.13 mg/L) [78]
V-48	**α-Terpineol**	Fumigant toxicity against *Lasioderma serricorne* (LC_{50} 3.27 mg/L air) [64]
V-65	**Citronellal**	Toxic against first instars larvae of Colorado potato beetle (% of mortality 100% after 96 h, 20 µl) [59]
V-73	**Cuminaldehyde**	Fumigant toxicity against Two-spotted spider mite, *Tetranychus urticae* (IC_{50} 0.31 mg/L) [78]
V-82	**Carvone**	Fumigant toxicity against Two-spotted spider mite, *Tetranychus urticae* (IC_{50} 1.28 mg/L) [78]
V-86	**(+)-Camphor**	Contact toxicity against *Lasioderma serricorne* (LC_{50} 13.4 µg/adults) adults [52] [64].<br>Fumigant toxicity against Two-spotted spider mite, *Tetranychus urticae* (IC_{50}) 7.72 mg/L [78], *T. pisana* (LC_{50} 7.98) and *S. littoralis* (LC_{50} 5.61 mg/L) [61] [65]
V-85	**ʟ-Fenchone**	Fumigant toxicity against Two-spotted spider mite, *Tetranychus urticae* (IC_{50} 3.65 mg/L) [78], *T. pisana* (LC_{50} 2.51 mg/L) and *S. littoralis* with (LC_{50} 2.27 mg/L) [61] [65]<br>Toxic against first instars larvae of Colorado potato beetle (% of mortality 100% after 48 h, 20 µl) [59]
V-92	**Thymol**	Fumigant toxicity against Two-spotted spider mite, *Tetranychus urticae* (IC_{50} 4.02 mg/L) [78]
V-98	**Dillapiole**	Active against nymphs and adults of *Diaphorina citri* [83]
V-106	**Cineole**	Fumigant toxicity against Two-spotted spider mite, *Tetranychus urticae* (IC_{50} 4.09 mg/L) [78], *T. pisana* (LC_{50} 4.17 mg/L) and *S. littoralis* (LC_{50} 4.34 mg/L) [65]<br>Toxic against first instars larvae of Colorado potato beetle (% of mortality 100% after 24 h, 20 µl) [59]
**Compounds with antiparasitic activity (Miscellaneous group)**		

No.	Compound	Activity [Ref.]
V-62	**β-Eudesmol**	Active against *Aedes aegypti* (LC_{50} > 50 µg/mL) [84]
V-91	**Estragole**	*In vivo* nematocidal potential in laboratory mice infected with *Heligmosomoides polygyrus* at 100 mg/kg body weight resulted in FECR reduction of 90.86% and TWCR reduction of 82.91% active as acetylcholinestrase inhibitor (IC_{50} of 0.176 mg/mL) for *H. scupense* and (IC_{50} 0.138 mg/mL) for *He. polygyrus* larvae [67].
V-94	**Eugenol**	Active against *Daphnia pulex* (LC_{50} 42.5 mg/mL) [24]
**Compounds with anti-scabies activity (effective against mite *Sarcoptes scabiei* mite)**		
V-39	Linalool	- *In vitro* miticidal efficacy (LT_{50} 39.9 h, at conc 0.5%) [85]<br>- Ovicidal activity against *Sarcoptes scabiei* eggs (EC_{50} 9.8%) [86]
V-41	Geraniol	- *In vitro* miticidal efficacy (LT_{50} 1.8 h, at conc 0.5%, LC_{50} values 0.91%, at 30 min) [85]<br>- Ovicidal activity against *Sarcoptes scabiei* eggs (EC_{50} 2.0%) [86]
V-48	α- Terpineol	-*In vitro* activity against *Sarcoptes scabiei* var *hominis* (LT_{50} 690 min, emulsifying ointment preparation, 0.15%) [87]
V-64	Citral	- *In vitro* miticidal efficacy (LT_{50} 6.1 h, at conc 0.5%) [85]<br>- Ovicidal activity against *Sarcoptes scabiei* eggs (EC_{50} 4.8%) [86]
V-94	Eugenol	- *In vitro* miticidal efficacy (LT_{50} 56.3 min, at conc 0.5%, LC_{50} values 0.79%, at 30 min) [85]<br>- Acaricidal activity against permethrin-sensitive scabies mites (EC_{50} 13.0 mM) and resistant mites (EC_{50} 40.7 mM) in contact bioassays [88]<br>- Ovicidal activity against *Sarcoptes scabiei* eggs (EC_{50} 0.9%) [86]
V-106	Cineole	- *In vitro* miticidal activity against *S. scabiei* var. *cuniculi* (LC_{50} 2.77 mg/mL, LT_{50} 3.6 h, 10% conc) [89]<br>- *In vitro* activity against *Sarcoptes scabiei* var *hominis* (LT_{50} 1020 min, emulsifying ointment preparation, 0.1%) [87]

EC_{50}: effective concentration causing 50% mortality; LC_{50}: lethal concentration causing 50% mortality; LT_{50}: lethal time to 50% mortality

Table S3: Volatile oil and naturally occurring volatile oil components with anti-scabies activity			
**No.**	**Essential oil name or volatile oil component**	**Natural Source**	**Anti-scabies activity**
1	Clove oil	Flower buds of *Eugenia aromatica* Linn, *Eugenia caryophyllus* Thumb)	*In vitro* activity against mites: 17.5 min (1%); 18.02 min (0.5%); 30.82 min (0.25); 63.06 min (0.25%) [90]<br>- *In vitro* acaricidal activity against permethrin-sensitive mites (15 min) and resistant mites (15 min) in contact bioassays (6.25%) [88]<br>-Contact bioassay: (10.0 min, 10% solution, 10.0 min 5%, 10 min, 1% soln.)<br>Fumigation bioassay: 5 min [91].
2	Nutmeg oil	Ripe seeds of *Myristica fragrance* Houtt	-*In vitro* acaricidal activity against permethrin-sensitive mites (15 min) and resistant mites (240 min) in contact bioassays (25%) [88].
3	Ylang Ylang oil	Flowers of *Cananga odorata*	- *In vitro* acaricidal activity against permethrin-sensitive mites (60 min) and resistant mites (300 min) in contact bioassays (25%) [88].
4	Cinnamon oil	Inner bark of *Cinnamomum zeylanicum*	-*In vitro* activity against mites<br>37.53 min (1%); 40.15 min (0.5 min)<br>42.19 min (0.25); 88.35 min (0.25 min) [90]
5	Tulsi oil	Aerial parts of *Ocimum sanctum*	-*In vitro* activity against mites<br>23.54 min (1%); 28.15 min (0.5 min)<br>42.64 min (0.25); 92.97 min (0.25 min) [90]
6	Lavander oil	Flower of *Lavandula angustifolia*	Contact bioassay: (20.0 min, 10% solution, 35.0 min 5%)<br>Fumigation bioassay: 5 min [91]
7	Tea tree oil (Melaleuca oil)	Leaves of *Melaleuca alternifolia*	Contact bioassay: (10.0 min, 10% solution, 30.0 min 5%)<br>Fumigation bioassay: 4 min [91]<br>Tea tree oil (emulsifying ointment preparation, 5%) 60 min [87]
8	Geranium oil	Aerial parts of *Pelargonium asperum*	Contact bioassay: (10.0 min, 10% solution, 20.0 min 5%)<br>Fumigation bioassay: 5 min [91]
10	Eucalyptus oil	Leaves of *Eucalyptus radiata*	Contact bioassay: (20.0 min, 10% solution, 150.0 min 5%)<br>Fumigation bioassay: 5 min [91]
11	Manuka oil	Leaves of *Leptospermum scoparium*	Contact bioassay: (30.0 min, 10% solution, 60.0 min 5%)<br>Fumigation bioassay: 23 min [91]
12	Bitter orange	Fruits of *Citrus aurantium amara*	Contact bioassay: (20.0 min, 10% solution, 50.0 min 5%)<br>Fumigation bioassay: 10 min [91]
13	Palmarosa oil	Leaves *Cymbopogon martinii*	Contact bioassay: (10.0 min, 10% solution, 10.0 min 5%, 20 min, 1% soln.)<br>Fumigation bioassay: 7 min [91]
14	Lippia oil	Leaves of *Lippia multiflora*	concentration of 20% v/v of the oil in a suitable formulation basis applied for 5 consecutive days may be sufficient for treating mite infestation [92]
15	Lemon grass oil	Leaves *Cymbopogon citratus*	*In vitro* miticidal activity (LC_{50} 1.37% after 1 h, LT_{50} 4.04 min at 10% conc.) and significant *in vitro* ovicidal activity [93]
16	Cardamomum oil	Fruits of *Elettaria Cardamomum* Maton.	*In vitro* miticidal activity (100% mortality, 10% concentration, 60 min, contact bioassay) [94]

Table S3: Volatile oil and naturally occurring volatile oil components with anti-scabies activity			
**No.**	**Essential oil name or volatile oil component**	**Natural Source**	**Anti-scabies activity**
17	Elsholtzia oil	Aerial paerts of *Elsholtzia densa* Benth	*In vitro* miticidal activity (LC_{50}: 7.678, 4.623, 2.543, 1.502, 1.298 and 0.981 mg/mL at 1, 2, 4, 8, 16, and 24 h, respectively; LT_{50} 45 min at 16 mg/mL) [95].
18	Lemon oil	Fruits of *Citrus lemon*	- *In vitro* miticidal activity at 20% conc. (88.6% mortality after 1 h treatment, 100% mortality after 24 h treatment)<br>- *In vitro* topical application of infected parts of rabbits showed that lemon oil 10 and 20% diluted in water caused mortality to 100% of mites after 24 h post application.<br>- *In vivo* application of 20% lemon oil on naturally infected rabbits showed complete recovery from clinical signs, absence of mite in microscopic examination from the second week of treatment [96].
19	Cedrus oil	Leaves and wood of *Cedrus deoduru*	Miticidal activity when applied topically to 24 lambs (3-6 months) naturally infected with *Sarcoptes* mites [97].
20	Terpinen-4-ol	Tea tree oil	- *In vitro* miticidal efficacy (LT_{50} 18.6 min, at conc 0.5%) [85]<br>- Terpinen-4-ol (2.1%) (emulsifying ointment preparation, 5%) 35 min [87]<br>- Ovicidal activity against *Sarcoptes scabiei* eggs (EC_{50} 5.1%) [86]
21	Isoeugenol	Cinnamon oil<br>Clove oil<br>ylang-ylang oil	- Acaricidal activity against permethrin-sensitive scabies mites (EC_{50} 24.6 mM) and resistant mites (EC_{50} 32.1 mM) in contact bioassays [88]
22	Acetyleugenol	Clove oil	- Acaricidal activity against permethrin-sensitive scabies mites (EC_{50} 19.4 mM) and resistant mites (EC_{50} 30.8 mM) in contact bioassays [88]
23	Carvacrol	Oregano oil<br>Origanum<br>Thyme oil	- *In vitro* miticidal efficacy (LT_{50} 1.03 min, at conc 0.5%, LC_{50} values 0.24%, at 30 min) [85]<br>- Ovicidal activity against *Sarcoptes scabiei* eggs (EC_{50} 0.5%) [86]

References

1. Chahal, K., et al., *Chemical composition and biological activity of Coriandrum sativum L.: A review.* 2017
2. Sayed-Ahmad, B., et al., *The Apiaceae: Ethnomedicinal family as source for industrial uses.* Industrial crops and products, 2017. **109**: p. 661-671https://doi.org/10.1016/j.indcrop.2017.09.027.
3. Ahmad, B.S., et al., *Fennel oil and by-products seed characterization and their potential applications.* Industrial crops and products, 2018. **111**: p. 92-98https://doi.org/10.1016/j.indcrop.2017.10.008.
4. Rożek, E., et al., *The chemical composition of the essential oil of leaf celery (Apium graveolens L. var. Secalinum Alef.) under the plants' irrigation and harvesting method.* Acta Scientiarum Polonorum. Hortorum Cultus, 2016. **15**(1): p. 149-159
5. Sahebkar, A. and M. Iranshahi, *Biological activities of essential oils from the genus Ferula (Apiaceae).* Asian Biomedicine, 2010. **4**(6): p. 835-847
6. Estekhdami, P. and A.N. Dehsorkhi, *Chemical Composition of Volatile Oil of Ferula assafoetida L.* International Journal of Research Studies in Agricultural Sciences, 2019. **5**: p. 9-14
7. Lončar, B., et al., *Do Climate Conditions Affect the Quality of the Apiaceae Fruits' Essential Oils?* Horticulturae, 2024. **10**(6): p. 5772024.
8. Miclea, V., et al., *Comparative study on essential oils of selected Apiaceous seeds cultivated in Transylvania.* Studia Ubb Chemia, 2019: p. 127-138
9. Foudah, A., *Comaparative Study of the Essential Oil, Phenol, Flavonoid contents and In Vitro Antioxidant Activity of Four Apiaceae Fruits.* INDO AMERICAN JOURNAL OF PHARMACEUTICAL SCIENCES, 2018. **5**(2): p. 1115-1121
10. Sowndhararajan, K., et al., *A review of the composition of the essential oils and biological activities of Angelica species.* Scientia pharmaceutica, 2017. **85**(3): p. 33
11. Swor, K., P. Satyal, and W.N. Setzer, *The Essential Oil Composition of Anthriscus caucalis M. Bieb.(Apiaceae) Growing Wild in Southwestern Idaho.* Natural Product Communications, 2023. **18**(7): p. 1-5https://doi.org/10.1177/1934578X231187699.
12. Asilbekova, D., et al., *Essential oil and lipids from leaves of Ferula kuhistanica.* Chemistry of Natural Compounds, 2019. **55**: p. 993-998DOI 10.1007/s10600-019-02877-3.
13. Badalamenti, N., et al., *The chemical composition of essential oil from Seseli tortuosum subsp. tortuosum and S. tortuosum subsp. maritimum (Apiaceae) aerial parts growing in Sicily (Italy).* Natural Product Research, 2023. **37**(20): p. 3519-3524doi: 10.1080/14786419.2022.2078819. .
14. Chizzola, R., *Essential oil composition of wild growing Apiaceae from Europe and the Mediterranean.* Natural Product Communications, 2010. **5**(9): p. 1477-1492
15. EL OTMANI, I.S., et al., *Chemical Composition and Antioxidant Activity of the Essential Oil of Wild Carrot Daucus carota L. from Morocco.* Journal of Chemical and Pharmaceutical Research, 2018. **10**(7): p. 30-34
16. Duman, A.D., et al., *Evaluation of bioactivity of linalool-rich essential oils from Ocimum basilucum and Coriandrum sativum varieties.* Natural Product Communications, 2010. **5**(6): p. 964-974https://doi.org/10.1177/1934578X1000500634.
17. De Carvalho, C.C. and M.M.R. Da Fonseca, *Carvone: Why and how should one bother to produce this terpene.* Food chemistry, 2006. **95**(3): p. 413-422Carvone: Why and how should one bother to produce this terpene.
18. Leal, S.M., et al., *Antiprotozoal activity of essential oils derived from Piper spp. grown in Colombia.* Journal of Essential Oil Research, 2013. **25**(6): p. 512-519https://doi.org/10.1080/10412905.2013.820669.
19. Moreno, É.M., et al., *Induction of programmed cell death in Trypanosoma cruzi by Lippia alba essential oils and their major and synergistic terpenes (citral, limonene and caryophyllene oxide).* BMC complementary and alternative medicine, 2018. **18**: p. 1-16https://doi.org/10.1186/s12906-018-2293-7.
20. Graebin, C.S., et al., *Synthesis and in vitro activity of limonene derivatives against Leishmania and Trypanosoma.* European journal of medicinal chemistry, 2010. **45**(4): p. 1524-1528https://doi.org/10.1016/j.ejmech.2009.12.061.
21. Tasdemir, D., et al., *Antiprotozoal activity of Turkish Origanum onites essential oil and its components.* Molecules, 2019. **24**(23): p. 4421https://doi.org/10.3390/molecules24234421.
22. Mikus, J., et al., *In vitro effect of essential oils and isolated mono-and sesquiterpenes on Leishmania major and Trypanosoma brucei.* Planta Medica, 2000. **66**(04): p. 366-368DOI: 10.1055/s-2000-8548.
23. Hachlafi, N.E., et al., *In vitro and in vivo biological investigations of camphene and its mechanism*

81. Feng, Y.-X., et al., *Efficacy of bornyl acetate and camphene from Valeriana officinalis essential oil against two storage insects.* Environmental Science and Pollution Research, 2019. **26**: p. 16157-16165https://doi.org/10.1007/s11356-019-05035-y.
82. Benelli, G., et al., *High toxicity of camphene and γ-elemene from Wedelia prostrata essential oil against larvae of Spodoptera litura (Lepidoptera: Noctuidae).* Environmental Science and Pollution Research, 2018. **25**: p. 10383-10391https://doi.org/10.1007/s11356-017-9490-7.
83. Volpe, H.X., et al., *Efficacy of essential oil of Piper aduncum against nymphs and adults of Diaphorina citri.* Pest Management Science, 2016. **72**(6): p. 1242-1249https://doi.org/10.1002/ps.4143.
84. Luna, E.C., et al., *Active essential oils and their components in use against neglected diseases and arboviruses.* Oxidative medicine and cellular longevity, 2019. **2019**(1): p. 6587150-6587202https://doi.org/10.1155/2019/6587150.
85. Li, M., et al., *In vitro efficacy of terpenes from essential oils against Sarcoptes scabiei.* Molecules, 2023. **28**(8): p. 3361-3368https://doi.org/10.3390/molecules28083361.
86. Li, M., et al., *Activity of terpenes derived from essential oils against Sarcoptes scabiei eggs.* Parasites & Vectors, 2021. **14**(1): p. 600-606https://doi.org/10.1186/s13071-021-05094-6.
87. Walton, S.F., et al., *Acaricidal activity of Melaleuca alternifolia (tea tree) oil: in vitro sensitivity of sarcoptes scabiei var hominis to terpinen-4-ol.* Archives of dermatology, 2004. **140**(5): p. 563-566doi:10.1001/archderm.140.5.563.
88. Pasay, C., et al., *Acaricidal activity of eugenol based compounds against scabies mites.* PloS one, 2010. **5**(8): p. 1-9https://doi.org/10.1371/journal.pone.0012079.
89. Hu, Z., et al., *In vitro acaricidal activity of 1, 8-cineole against Sarcoptes scabiei var. cuniculi and regulating effects on enzyme activity.* Parasitology research, 2015. **114**: p. 2959-2967 https://doi.org/10.1007/s00436-015-4498-8.
90. Andriantsoanirina, V., et al., *In vitro efficacy of essential oils against Sarcoptes scabiei.* Scientific Reports, 2022. **12**(1): p. 7176-7184https://doi.org/10.1038/s41598-022-11176-x.
91. Fang, F., et al., *In vitro activity of ten essential oils against Sarcoptes scabiei.* Parasites & vectors, 2016. **9**: p. 1-7DOI 10.1186/s13071-016-1889-3.
92. Oladimeji, F., et al., *Pediculocidal and scabicidal properties of Lippia multiflora essential oil.* Journal of Ethnopharmacology, 2000. **72**(1-2): p. 305-311https://doi.org/10.1016/S0378-8741(00)00229-4.
93. Li, M., et al., *Lemongrass (Cymbopogon citratus) oil: A promising miticidal and ovicidal agent against Sarcoptes scabiei.* PLoS Neglected Tropical Diseases, 2020. **14**(4): p. 1-10https://doi.org/10.1371/journal.pntd.0008225.
94. Sharma, B., N. Vasudeva, and S. Sharma, *Chemical composition and anti-scabies activity of essential oil of Elettaria cardamomum Maton. leaves.* ACTA Pharmaceutica Sciencia, 2020. **58**(2): p. 192-203DOI: 10.23893/1307-2080.APS.05812.
95. Zhou, Y., et al., *Composition and acaricidal activity of essential oil from Elsholtzia densa Benth against Sarcoptes scabiei mites in vitro.* Veterinární medicína, 2019. **64**(4): p. 178-183https://doi.org/10.17221/20/2018-VETMED.
96. Aboelhadid, S., et al., *In vitro and in vivo effect of Citrus limon essential oil against sarcoptic mange in rabbits.* Parasitology research, 2016. **115**: p. 3013-3020https://doi.org/10.1007/s00436-016-5056-8.
97. Sharma, D., et al., *Evaluation of oil of Cedrus deodara and benzyl benzoate in sarcoptic mange in sheep.* Small Ruminant Research, 1997. **26**(1-2): p. 81-85https://doi.org/10.1016/S0921-4488(97)00002-3.
